# *TROPPO*: tissue-specific reconstruction and phenotype prediction using omics data

**DOI:** 10.1093/bioadv/vbaf113

**Published:** 2025-05-19

**Authors:** Alexandre Oliveira, Jorge Ferreira, Vítor Vieira, Bruno Sá, Miguel Rocha

**Affiliations:** Centre of Biological Engineering, University of Minho, Braga 4710-057, Portugal; Centre of Biological Engineering, University of Minho, Braga 4710-057, Portugal; Centre of Biological Engineering, University of Minho, Braga 4710-057, Portugal; Centre of Biological Engineering, University of Minho, Braga 4710-057, Portugal; Centre of Biological Engineering, University of Minho, Braga 4710-057, Portugal; LABBELS—Associate Laboratory, Braga 4710-057, Portugal

## Abstract

**Summary:**

The increasing availability of high-throughput technologies in systems biology has advanced predictive tools like genome-scale metabolic models. Despite this progress, integrating omics data to create accurate, context-specific metabolic models for different tissues or cells remains challenging. A significant issue is that many existing tools rely on proprietary software, which limits accessibility. We introduce TROPPO, an open-source Python library designed to overcome these challenges. TROPPO supports a wide range of context-specific reconstruction algorithms, provides validation methods for assessing generated models, and includes gap-filling algorithms to ensure model consistency, integrating well with other constraint-based tools.

**Availability and implementation:**

TROPPO is implemented in Python and is freely available at https://github.com/BioSystemsUM/TROPPO and https://pypi.org/project/TROPPO/.

## 1 Introduction

In recent years, the synergy between biology and informatics has increasingly revealed the intricacies of cellular processes, from genome sequencing to reconstructing metabolic pathways in human cells. Advances in high-throughput technologies have expanded the scientific community’s ability to analyze cells as complex, interconnected systems. Genome-scale metabolic models (GSMMs), derived from integrating genome data into Constraint-Based Models (CBMs), are pivotal in predicting cellular behaviors tied to metabolite consumption and production rates (Fang *et al.* 2020).

Over the years, numerous bacterial GSMMs have been developed. Despite the inherent complexity, significant progress has also been made in creating generic metabolic models for more complex organisms, such as plants (Cunha *et al.* 2023, Sampaio *et al.* 2024) and humans (Brunk *et al.* 2018, Robinson *et al.* 2020). Although several of these models are available, they often encompass the entire metabolism of the organism and may not account for metabolic variability across different cell types, leading to potentially incomplete predictions. Furthermore, even bacterial models are influenced by environmental conditions; using a model outside its validated conditions can result in inaccurate outcomes. This underscores the importance of developing context- and tissue-specific models to enhance prediction accuracy (Vieira *et al.* 2022).

One of the key advantages of GSMMs is their ability to seamlessly integrate omics data, enabling further model refinement to replicate specific conditions. Over the past decades, numerous algorithms have been developed to tailor generic models for specific tissues or cell types, thereby facilitating context-specific phenotype predictions and analyses (Estévez and Nikoloski 2014). Omics integration algorithms can be categorized into three groups based on their methodological objectives: GIMME-like, iMAT-like, and MBA-like approaches. GIMME-like algorithms ensure that the predicted *in silico* phenotype aligns with experimentally observed data while maintaining the activity of predefined essential metabolic functions. Similarly, iMAT-like algorithms, such as iMAT and INIT, prioritize flux similarity to experimental data but do not impose predefined metabolic functions, making them more accessible for use. In contrast, MBA-like methods focus on ensuring model consistency, generating context-specific models that are free from blocked reactions (Estévez and Nikoloski 2014). Examples of such methods include FASTcore, CORDA, and SwiftCore.

While a variety of algorithms exist for this purpose, they are implemented across different platforms. For instance, some of the most relevant methods are available through the COBRA toolbox, which relies on the commercial MATLAB environment (Heirendt *et al.* 2019). COBRApy, a Python implementation of this tool exists; however, it does not include any omics integration method (Ebrahim *et al.* 2013). MEWpy (Pereira *et al.* 2021), on the other hand, introduced GIMME (Becker and Palsson 2008), iMAT (Zur *et al.* 2010), and e-Flux (Colijn *et al.* 2009) in a Python-based environment; however, it still lacks a broad range of other relevant methods.

In this work, we introduce Tissue-specific RecOnstruction and Phenotype Prediction using Omics data (TROPPO), an open-source software platform developed in Python. TROPPO is designed to implement a broad range of omics integration algorithms for GSSMs, allowing users to input a generic model along with various types of omics data to generate context- or tissue-specific models and/or phenotype predictions. Existing tools for omics-based metabolic model reconstruction often require proprietary software, such as MATLAB-based COBRA Toolbox, which restricts accessibility and reproducibility. TROPPO addresses these challenges by offering a fully open-source, Python-based framework that consolidates multiple reconstruction algorithms, validation procedures, and gap-filling methods, ensuring flexibility, usability, and broader accessibility for the research community.

## 2 Architecture of TROPPO

**Figure 1.**
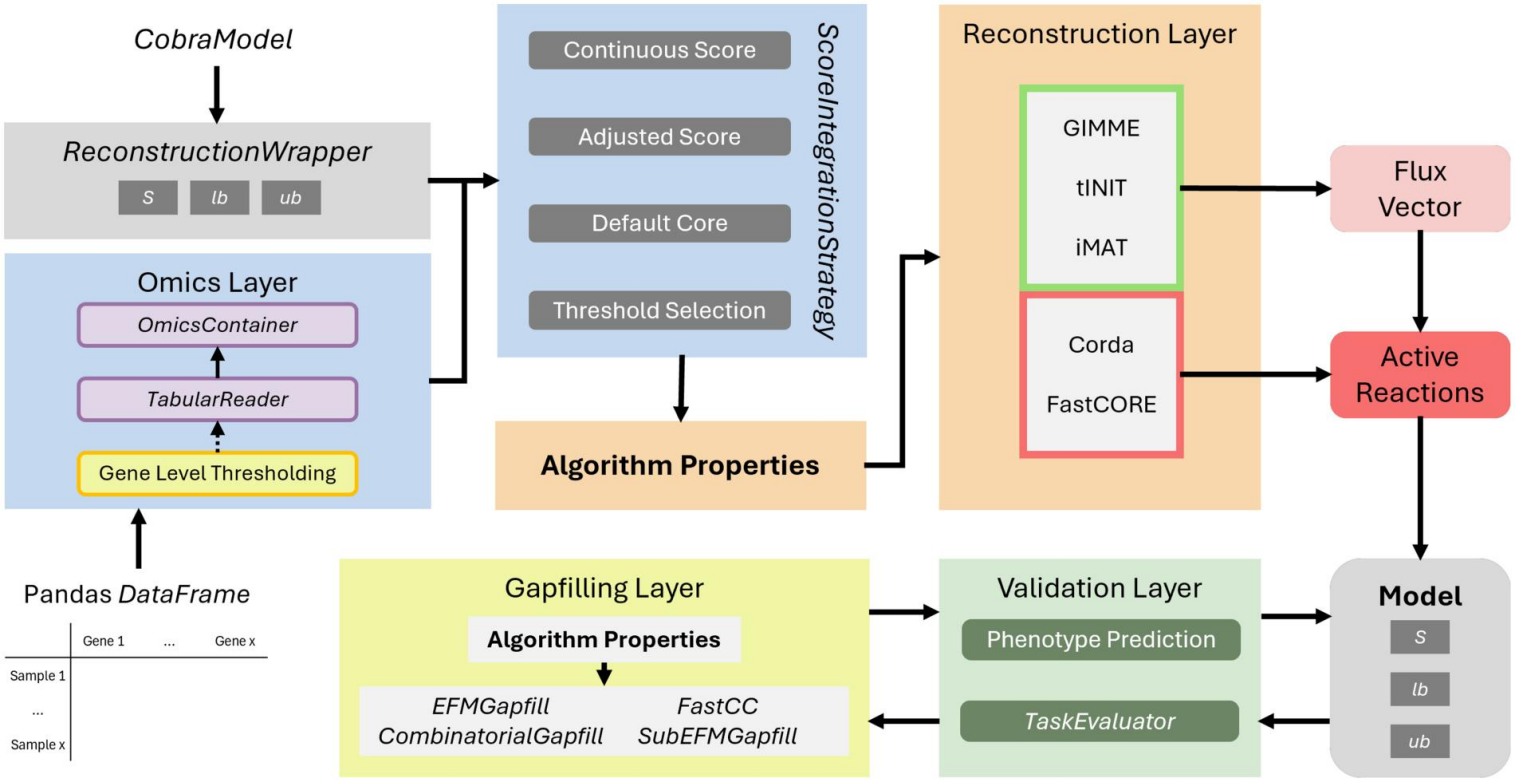
TROPPO’s pipeline starts with instantiating a generic model using the ReconstructionWrapper class. Omics data are then loaded into an OmicsContainer instance. Optional gene-level thresholding can convert gene expression levels into confidence scores. TROPPO then performs reconstruction using integrated scores and specific algorithm parameters, returning active reactions. Inactive reactions are removed, and gap-filling methods address any missing essential reactions.

TROPPO is a Python-based modular framework for context-specific metabolic models reconstruction, as illustrated in Fig. 1. It integrates with external tools like COBRApy (Ebrahim *et al.* 2013) and ReFramed (Machado *et al.* 2023) for model reading and manipulation and relies on the CoBAMP library (Vieira and Rocha 2019) and optlang (Sonnenschein *et al.* 2018) for constraint-based pathway analysis and solver connectivity. The framework comprises four main components: an omics data processing layer for transcriptomics and proteomics, a modular reconstruction layer for omics integration methods, a validation layer, and a gap-filling layer for model refinement.

- **Omics Layer**: This layer provides methods for managing omics data, including loading tabular datasets with the TabularReader and converting each sample into OmicsContainer instances. It supports the calculation of reaction scores using four integration methods: Continuous score, Adjusted score, Default core, and Threshold Selection. Additionally, it allows for the calculation of confidence scores for enzyme activity through GeneLevelThresholding.
- **Reconstruction Layer**: For this layer, the input consists of a model wrapper and the results from the integration scoring methods. TROPPO currently implements five reconstruction algorithms: GIMME (Becker and Palsson 2008), tINIT (Agren *et al.* 2014), iMAT (Zur *et al.* 2010), FastCORE (Vlassis *et al.* 2014), SwiftCore (Tefagh and Boyd 2020), and CORDA (Schultz and Qutub 2016). Each algorithm requires a specific set of parameters, which must be defined and provided as input. The final output of this layer is a set of reactions deemed active based on the omics data.
- **Validation Layer**: After removing the inactive reactions, the model can be validated by assessing the metabolic phenotype with using ContextSpecificModelSimulator or by determining if the model can perform a set of specified metabolic tasks with TaskEvaluator.
- **Gap-filling Layer**: Addresses missing reactions with algorithms like Elementary Flux Modes (EFM), SubEFM, FastCC, and Combinatorial gap-fill.

Overall, TROPPO aims to provide a comprehensive framework for context-specific model reconstruction and validation, which, apart from MEWpy, was lacking in a Python environment (Table 1). Detailed descriptions of these layers and methods are available in Supplementary File S1. Furthermore, descriptions of all implemented integration methods, as well as validation and benchmarking details, are available in Supplementary File S2.

**Table 1.** Comparison of TROPPO with four widely used tools for constraint-based modeling—RAVEN2, COBRA Toolbox, COBRApy, and MEWpy—across various functionalities related with omics data integration and context-specific model reconstruction.^a^

Feature		RAVEN 2	COBRA Toolbox	COBRApy	MEWpy	Troppo
Integration Methods	FastCORE		X			X
	CORDA					X
	GIMME		X		X	X
	tINIT	X	X			X
	SwiftCore		X			X
	iMAT		X		X	X
	Mba		X			
	Eflux		X		X	
	Moomin		X			
Simulation Methods	FBA	X	X	X	X	X
	pFBA		X	X	X	
	FVA		X	X	X	
	MOMA	X	X	X	X	
	ROOM		X	X	X	
Gapfilling Methods		X	X	X		X
Task Evaluation		X				X
Transcript Activity Scores						X

a An “X” denotes that a specific feature is available in the corresponding tool.

## 3 Examples and documentation

TROPPO provides a range of integration algorithms for creating context-specific models through a straightforward process: (i) load a model and create a ReconstructionWrapper instance, (ii) import omics data into an OmicsContainer, (iii) compute integrated scores for reactions, and (iv) apply the chosen integration algorithm. For a simplified approach, the run_from_omics method in the ReconstructionWrapper class is available. Detailed tutorials and documentation are accessible at https://troppo-bisbi.readthedocs.io/ and https://github.com/BioSystemsUM/troppo/tree/master/tests.

In addition, two case studies are detailed in the Supplementary Files. The first (Supplementary File S3) involves reconstructing 320 context-specific models for the MCF-7 cell line. These samples were obtained from the Cancer Cell Line Encyclopedia (CCLE) (Nusinow *et al.* 2020) and integrated into the HumanGEM model (Robinson *et al.* 2020) using the FastCORE and tINIT algorithms, a wide variety of combinations of parameters and thresholding combinations to calculate transcript activity scores (TASs). The pipeline followed in this example was published by Vieira *et al.* (2022) and can be consulted for further details. The files for this case study are available on *Troppo’s* GitHub page (https://github.com/BioSystemsUM/troppo/tree/master/examples/mcf7_case_study).

The second (Supplementary File S4) utilized transcriptomics data from Desai *et al.* (2020) (GEO: GSE150316), which included samples from 23 infected patients across various tissues, supplemented with healthy samples from the Genotype-Tissue Expression (GTEx) Portal Consortium (2020) (GTExConsortium 2020). These data were also integrated into the HumanGEM model (Robinson *et al.* 2020) using FastCORE. This pipeline was part of a work submitted to the 10th IFAC International Conference on Foundations of Systems Biology in Engineering (FOSBE 2024) (Oliveira *et al.* 2024). The notebooks and data used for this case study are also available in TROPPO’s GitHub page (https://github.com/BioSystemsUM/troppo/tree/master/examples/covid19_case_study).

## 4 Application examples

Since TROPPO’s release, several other studies have utilized the package. The first publication, by Barata *et al.* (2022), employed TROPPO’s tINIT and FastCORE implementations to reconstruct context-specific models of cancer stem cells across different tissues. Subsequently, TROPPO has been used a multi-tissue model of *Quercus suber* (Cunha *et al.* 2023), cell line–specific GSSMs for various NCI-60 cell lines (Barata *et al.* 2024), and a diel multi-tissue model of *Vitis vinifera* (Sampaio *et al.* 2024). Additionally, TROPPO has been integrated into COMO, which is a pipeline for drug target identification that utilize metabolic models and omics data (Bessell *et al.* 2023).

## 5 Conclusion

TROPPO represents a step forward in the field of Python-based context-specific metabolic modeling, offering a comprehensive and flexible framework for reconstructing and analyzing tissue- and condition-specific GSMMs. By integrating multiple omics data types, reconstruction algorithms, and validation methodologies, TROPPO enables researchers to generate accurate and biologically meaningful models tailored to specific experimental conditions. Its modular Python-based architecture facilitates compatibility with widely used tools such as *COBRApy*, while also allowing easy expansion to incorporate novel methods and expanded support for additional data types.

The versatility of TROPPO is demonstrated through its application to diverse use cases, ranging from cancer cell line modeling to multi-tissue plant and human disease studies. Its ability to seamlessly integrate omics data and support multiple reconstruction algorithms ensures broad applicability across a wide array of research domains. Moreover, the package’s task evaluation and phenotype prediction modules enable detailed assessments of metabolic phenotypes and validation of the generated models, making it a meaningful tool in systems biology.

## Supplementary Material

vbaf113_Supplementary_Data
