# Supplementary material for: TROPPO: tissue-specific reconstruction and phenotype prediction using omics data
Source: Bioinform Adv. 2025 May 19;5(1):vbaf113. doi: 10.1093/bioadv/vbaf113 (PMC12179386; doi:10.1093/bioadv/vbaf113)
Supplement: vbaf113_Supplementary_Data [file vbaf113_supplementary_data.zip › Supplementary_File_1.pdf]

Supplementary File 1

TROPPO Framework: Implementation Overview

Alexandre Oliveira[1,*], Jorge Ferreira[1,*], Vítor Vieira[1,*], Bruno Sá[1], Miguel Rocha[1,2,†]

[1]CEB - Centre of Biological Engineering, University of Minho, 4710-057 Braga, Portugal
[2]LABBELS – Associate Laboratory, Braga/Guimarães, Portugal

[†]Corresponding author: mrocha@di.uminho.pt ,
[*]These authors contributed equally for this work.

TROPPO is a novel library implementing several context-specific model extraction methods, essential for modelling human metabolism through omics data integration, in the Python programming language. This collaborative effort was necessary since many of these methods are available only through MATLAB, either as a part of the COBRA Toolbox or as separate scripts. The library can be divided in three main parts, namely, algorithms, omics data processing and model validation. The source code for TROPPO can be found on GitHub at `https://github.com/BioSystemsUM/troppo`.

Omics data platform: TROPPO implements features to process data with transcriptomics and proteomics measurements. It provides simple commands to import the data through the pandas library [3], an external Python package that assists in loading and manipulating tabular data from various formats. Omics measurements are standardised into data structures that function as inputs for the algorithms implemented in TROPPO. Identifier conversion features are also provided and genes present on the HUGO Gene Nomenclature Committee (HGNC) database can be easily converted into different nomenclatures.

Context-specific model reconstruction: The main feature of TROPPO is the implementation of context-specific metabolic reconstruction (CSMR) algorithms in a Python environment. Several context-specific model extraction methods were reimplemented as a part of this framework in a modular architecture, where new algorithms can easily be added, using standardised inputs. CoBAMP is used for these implementations and thus, TROPPO also shares the same compatibility with other frameworks. Model refinement is also achieved through gap filling methods.

Model validation: The package also provides methods to validate context-specific models, leveraging the efficient batch simulation routines provided by CoBAMP to predict phenotypes for multiple models and biological scenarios with simple commands. Moreover, TROPPO also includes a task evaluation module and with an intuitive metabolic task definition. Tasks can also be loaded and exported into JavaScript Object Notation (JSON) files for later use.

1. Omics data processing

The class architecture for omics data handling in TROPPO is based on previous work by Correia et al. [2]. The omics module contains the data structures and routines required to load and process omics datasets. The main classes and data flow in the omics module are represented in Figure 1.

`OmicsContainer`: This class serves as a generic container for storing omics data samples. Each `OmicsContainer` instance has four main attributes:

- `data`: A dictionary mapping valid biological database identifiers with a numeric value;

- `condition`: A string identifying the sample;
- `nomenclature`: A string identifying the biological database to which the identifiers belong;
- `omicstype`: A string identifying whether the data quantifies transcriptomics, proteomics or metabolomics.

This object contains several methods to handle missing values, apply transformations, and extract meaningful sets of genes or metabolites based on a user-defined threshold. For transcriptomics data, it allows gene/transcript names to be converted into a desired nomenclature.

`get_integrated_data_map`: This is the most important method added to the `OmicsContainer` class, responsible for generating `OmicsDataMap` objects containing reaction scores that can be integrated into constraint-based models. It requires an `AbstractModelObjectReader` instance from CoBAMP, which is used to match the model's gene identifiers and gene-protein-reaction (GPR) rules with those present in the `OmicsContainer` data field. After processing, this method returns an `OmicsDataMap` object with integrated scores.

`TabularContainer`: Since most omics datasets are stored as two-dimensional arrays, this class was implemented to facilitate the loading and storage of measurements along with their associated biological entity and sample identifiers.

`OmicsMeasurementSet`: This subclass extends `TabularContainer` by allowing dataset entries to be converted into `OmicsContainer` objects.

`TypedOmicsMeasurementSet`: This subclass of `OmicsMeasurementSet` further extends functionality by enabling the conversion of feature identifiers when provided with an `IdentifierMap` object. The `IdentifierMap` is capable of converting biological identifiers for various databases when supplied with a mapping from resources such as HGNC [5].

`ScoreIntegrationStrategy`: This abstract class defines a template for integrating `OmicsDataMap` objects as inputs for context-specific model reconstruction algorithms. It enforces an `integrate` method that takes an `OmicsDataMap` object as input and returns an appropriate score format based on the algorithm being used. Five integration strategies were implemented as part of the context-specific metabolic reconstruction pipeline in this study.

`GeneLevelThresholding`: This sub-module offers an alternative approach for assessing activity at the gene level by using transcript activity scores (TAS). The method, introduced by Richelle et al. [4], includes three thresholding strategies:

- Global thresholding
- Local T1 thresholding
- Local T2 thresholding

All three strategies are implemented in the `GeneLevelThresholding` sub-module and will be described next.

1.1. Calculating transcript activity scores

In RNA-seq-based metabolic model integration, gene expression levels are used to infer enzyme activity by mapping genes to reactions through GPR rules. Several methods for calculating TAS have been proposed by Richelle et al. (2019) [4], which vary in thresholding strategies. This implementation focuses on the different thresholding approaches, assuming a linear relationship between mRNA and protein levels. A global thresholding method first determines a collective expression cutoff across all genes to classify transcripts as inactive or active with high confidence. Additionally, a local thresholding method evaluates each gene independently, assigning unique thresholds based on its expression distribution across samples. Quantiles (10%, 25%, 50%, 75%,

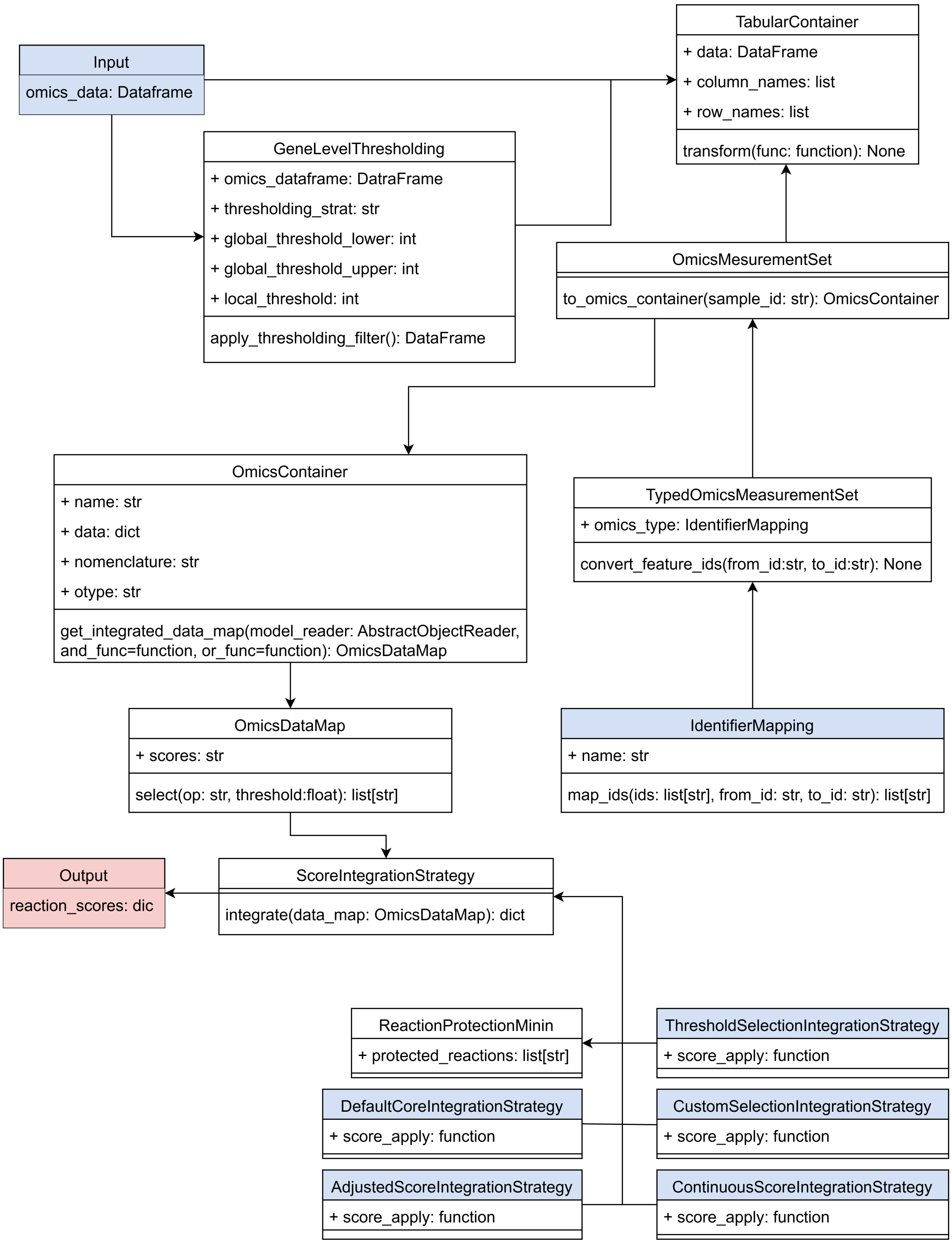

Figure 1. Overview of the omics data processing layer implemented in *troppo*. The required inputs (highlighted in blue) include a pandas DataFrame containing the omics data, an `IntegrationStrategy`, and optionally, an `IdentifierMapping` if the gene IDs in the dataset do not match those in the generic model. As output (highlighted in red), the omics layer returns a dictionary with the scores for each reaction.

and 90%) are used to derive multiple local thresholds, and their mean is used to establish a representative global threshold.

Three scoring strategies are implemented: Global, Local T1, and Local T2. The Global method classifies transcripts as active if their expression surpasses a predefined upper threshold (GTU). Local T1 refines this by requiring a transcript to exceed both the GTU and a gene-specific local threshold (LT). Local T2 introduces an additional lower global threshold (GTL), allowing transcripts with intermediate expression levels to be classified based on their local threshold. After thresholding, TAS values are assigned, with negative values indicating inactivity and positive values signifying active transcripts. This refined approach enhances transcriptomic data integration into metabolic reconstructions, improving phenotype prediction and downstream analysis.

2. Context-specific reconstruction and validation methods

Context-specific model reconstruction methods in TROPPO are organized into two distinct modules. The `methods` module contains algorithm implementations that operate independently of metabolic model abstractions and primarily process inputs in numerical formats. Additionally, the `methods_wrappers` module includes classes and mappings that allow these algorithms to interact with higher-level data structures, such as objects representing entire metabolic models.

2.1. Model extraction and refinement algorithms

The methods module in TROPPO contains algorithms that directly operate on constraint-based models or utilize linear programming formulations based on network topology and stoichiometry. The class structure and modular organization of these algorithms are visualized in Figure 2.

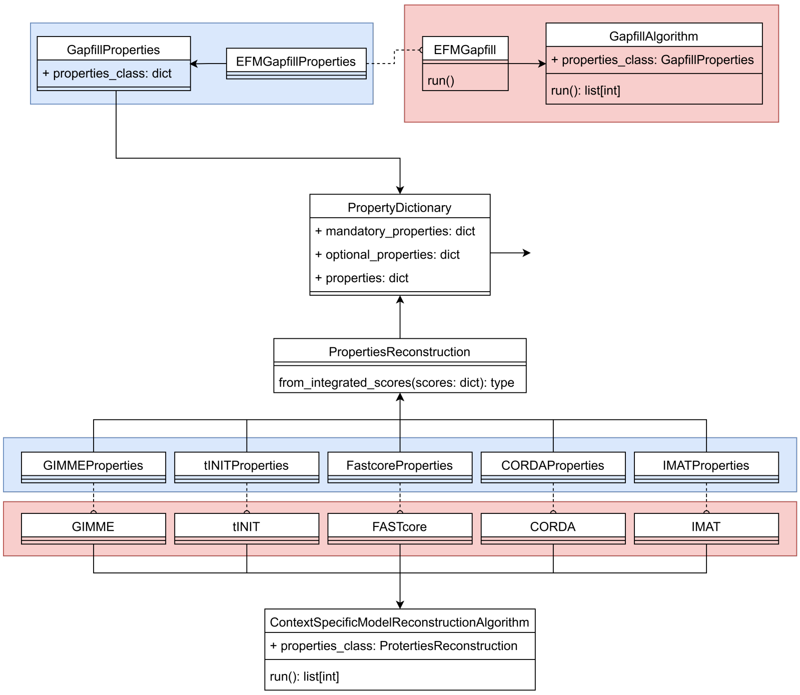

Figure 2. Overview of the class structure for model extraction and refinement methods in TROPPO. Property classes for reconstruction and gap-filling algorithms are highlighted in blue, while implementation classes are shown in red.

`ContextSpecificModelReconstructionAlgorithm` and `GapfillAlgorithm` are wrappers that share a common constructor template. Both require a stoichiometric matrix, flux bound vectors,

and an object containing algorithm-specific properties. The mandatory `run` class method outputs a Boolean vector, where each flux in the input model is classified as:

- **False**: Reaction is absent in the reconstructed model.
- **True**: Reaction is present in the reconstructed model for the given context.

The supplied context is passed as a property within the appropriate class.

`PropertiesReconstruction` is a subclass of CoBAMP's `PropertyDictionary` and defines a single mandatory property: a string that specifies the linear programming solver to be used for optimizations. By defining omics data input as a property, this class ensures compatibility across different algorithms while decoupling input processing from model extraction.

`GapfillProperties` is an extension of `PropertiesReconstruction` that includes additional optional properties:

- `lsystem_args`: A parameter influencing the steady-state balance of the model, useful in task-based gap filling.
- `avbl_fluxes`: A list of fluxes considered present prior to executing the gap-filling algorithm.

`PropertiesReconstruction` and `GapfillProperties` instances encapsulate a dictionary that maps property identifiers to their respective values. This implementation allows for the definition of both mandatory and optional properties, as well as type checking. Every algorithm in TROPPO requires an appropriate subclass implementation to accommodate specific input requirements.

2.2. Context-Specific Method Wrappers

With a generic definition of a context-specific model extraction algorithm, the entire pipeline can be accessed through a higher-level wrapper class (Figure 3). This wrapper includes routines for handling an `AbstractModelObjectReader`, an `OmicsContainer`, and algorithm-specific properties to return the algorithm's output.

- `map_properties_algorithms`: Maps `PropertyDictionary` subclasses to their corresponding `ContextSpecificModelReconstructionAlgorithm` subclasses.
- `algorithm_instance_map`: Maps algorithm names, represented as strings, to their appropriate `ContextSpecificModelReconstructionAlgorithm` subclass.
- `integration_strategy_map`: Maps scoring strategy names, represented as strings, to their corresponding `ScoreIntegrationStrategy` from the `omics.integration` module.

`ModelBasedWrapper`: This class is responsible for extracting the appropriate inputs from an `AbstractModelObjectReader`. It retrieves key elements such as the stoichiometric matrix and flux bound vectors, which are necessary for creating algorithm instances. Subclasses of this abstract class must implement `run` methods that interpret `PropertyDictionary` subclass instances and associate their type with the correct algorithm.

`methods_wrappers module`: This module maintains several dictionaries that store mappings between algorithm properties and their corresponding reconstruction algorithms. These dictionaries are continuously updated as new methods are implemented:

`ReconstructionWrapper`: This class is a subclass of `ModelBasedWrapper` and provides the `run_from_omics` method. Its mandatory arguments include:

- `omics_data`: An `OmicsContainer` instance containing loaded data, an iterable with numeric scores for each reaction, or a dictionary mapping reaction identifiers to their activity scores.

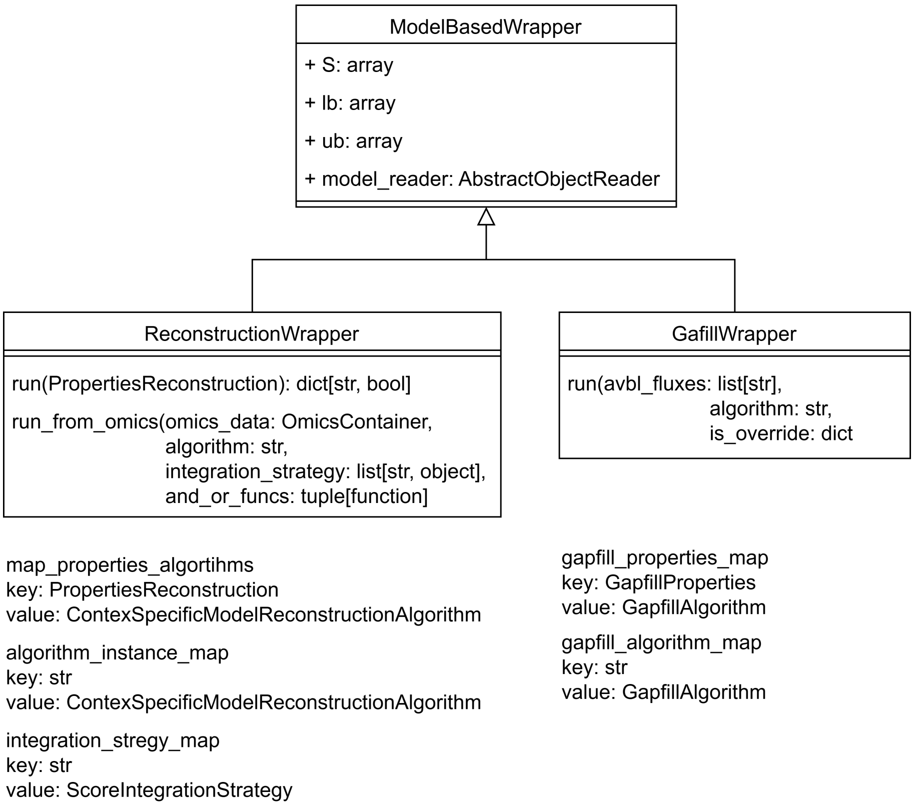

Figure 3. Overview of the class structure for the higher-level wrappers used to simplify the process of reconstructing and refining models with TROPPO.

- `algorithm`: A string representing an algorithm found in `algorithm_instance_map`.
- `integration_strategy`: A `ScoreIntegrationStrategy` instance or a tuple containing the integration strategy name (from `integration_strategy_map`) and its required parameters.
- `and_or_funcs`: A tuple with two functions to replace the AND and OR Boolean operators, respectively.

The output of this method is a dictionary mapping reaction identifiers to Boolean values, indicating their presence or absence in the reconstructed model.

`GapfillWrapper`: A similar class architecture is used for gap-filling methods. This class implements a `run` method with the same input parameters as those in `GapfillProperties`, along with an algorithm string that specifies the gap-filling algorithm. These algorithms are stored in `gapfill_algorithm_map`, which maps algorithm names to their corresponding classes.

3. Model Validation

Model validation in TROPPO can be performed either through simulation using a phenotype prediction method (available in the `validation` sub-module) or through metabolic tasks (handled by the `tasks` module).

3.1. Phenotype Prediction

`ContextSpecificModelSimulator`: This is the only simulation method implemented in TROPPO, designed to provide an abstraction for flux-bound contexts derived from context-specific model

extraction methods and environmental conditions. While constraint-based model simulation tools such as COBRApy, ReFramed, and CoBAMP already exist, `ContextSpecificModelSimulator` simplifies integration within the TROPPO framework. This method requires three inputs:

- `ConstraintBasedModelSimulator`: This class serves as the underlying simulation engine from the CoBAMP framework. It allows users to run simulations across different environmental conditions by applying predefined flux constraints.
- `scenarios dictionary`: This dictionary maps environmental condition names to dictionaries containing flux identifiers and their corresponding upper and lower bounds. It serves as a structured input for defining context-specific metabolic constraints.

`post_process`: This optional argument in `ContextSpecificModelSimulator` allows users to specify a function for processing the simulation results into a desired output format.

`simulate`: Similar to the `batch_simulate` method in `ConstraintBasedModelSimulator`, the `simulate` method requires:

- Objective coefficients
- A simulation function (phenotype prediction method)
- A `contexts` dictionary, which maps sample names to outputs from `ReconstructionWrapper`'s `run_from_omics` method. This output consists of dictionaries mapping flux identifiers to Boolean values, indicating their presence or absence in the reconstructed model.

`batch_simulate`: This method is used by the `simulate` method to parallelize the optimization process, ensuring efficient computation across multiple simulation scenarios.

3.2. Task Evaluation

Metabolic task evaluation is a feature present in both the COBRA and RAVEN toolboxes for MATLAB but is absent in Python frameworks. Due to differences in metabolic task definitions across these platforms, a generic data structure was developed in TROPPO for loading and evaluating tasks.

`tasks module`: This module includes core components for defining, evaluating, and managing metabolic tasks. It contains three key classes:

- `Task`: Stores the metabolic task definition.
- `TaskEvaluator`: Uses CoBAMP to evaluate metabolic tasks in parallel.
- `TaskIO`: Handles reading and writing of tasks in JSON and Microsoft Excel formats.

`TaskIO`: This abstract class unifies parsing and writing functions for metabolic tasks. It requires the implementation of `read_from_string` and `write_to_string`, which respectively accept a string and a task as input, returning either a `Task` object or a formatted string. This class also implements the `read_task` and `write_task` methods, which call the above functions to handle task input/output according to the specified format. JSON is the primary format for reading and storage due to its compatibility with Python data structures. However, a Microsoft Excel parser is also available since most existing task lists are in this format.

`Task`: The central class in this module, it defines a metabolic task as a set of flux conditions that modify an existing `ConstraintBasedModel` instance from CoBAMP to assess a metabolic function. Based on the metabolic task definitions by Thiele, Agren, and Richelle [6, 1, 4], a `Task` object may contain the following inputs:

- `name`: A string representing the task's name.
- `annotations`: A dictionary containing optional metadata such as extended descriptions.
- `should_fail`: A Boolean flag indicating whether the task is expected to fail.
- `reaction_dict`: A dictionary mapping new reaction names to stoichiometric coefficients for each metabolite, along with numerical lower and upper bounds.
- `flow_dict`: A dictionary mapping metabolite names to their lower and upper bounds for extracellular interactions. This is further divided into two separate dictionaries:
  - `inflow_dict`: Defines uptake fluxes.
  - `outflow_dict`: Defines secretion fluxes.
- `mandatory_activity`: A list of reactions that are required to be active when evaluating the task. While this does not affect evaluation directly, it provides additional constraints for analysis.

The `Task` object also contains auxiliary methods for manipulating reaction identifiers and evaluating previously determined flux distributions. Although it provides methods for evaluating a single task on a `ConstraintBasedModel` object, its primary function is to store the task's definition and specify how the metabolic model should be modified for evaluation.

`get_add_reaction_cmds`: This method utilizes partial functions for queuing calls to reaction addition functions in `ConstraintBasedModel`. It ensures systematic addition of new reactions before evaluation.

`get_task_bounds`: This function returns a dictionary that maps flux identifiers to the corresponding flux bounds that need to be modified for the task. This allows for dynamic adjustments of metabolic constraints based on task specifications.

`TaskEvaluator`: This class allows the evaluation of metabolic tasks by instantiating a `Task` object alongside a `ConstraintBasedModel` or `AbstractModelObjectReader`. It utilizes a CoBAMP metabolic model as the foundation for optimization procedures.

`current_task`: Since `TaskEvaluator` loads all provided tasks but evaluates only one at a time, this class attribute holds a string corresponding to the task's name. Users can modify this attribute to switch between tasks dynamically.

`batch_evaluate`: For large sets of flux bounds, this function enables parallel evaluation of metabolic tasks. Although it does not provide true parallel execution, it significantly enhances performance by reducing redundant computations.

The evaluation result is returned as a tuple containing three elements:

- Task evaluation status relative to the expected outcome (based on the `should_fail` parameter).
- A dictionary mapping flux identifiers in the `mandatory_activity` parameter to their activation status (active or inactive).
- The flux distribution generated during the task evaluation.

References

[1] Rasmus Agren, Sergio Bordel, Adil Mardinoglu, Natapol Pornputtapong, Intawat Nookaew, and Jens Nielsen. Reconstruction of genome-scale active metabolic networks for 69 human cell types and 16 cancer types using init. *PLoS computational biology*, 8(5):e1002518, 2012.

[2] Sara Correia and Miguel Rocha. A critical evaluation of methods for the reconstruction of tissue-specific models. In *Progress in Artificial Intelligence: 17th Portuguese Conference on Artificial Intelligence, EPIA 2015, Coimbra, Portugal, September 8-11, 2015. Proceedings 17*, pages 340–352. Springer, 2015.

[3] The pandas development team. pandas-dev/pandas: Pandas, February 2020.

[4] Anne Richelle, Austin WT Chiang, Chih-Chung Kuo, and Nathan E Lewis. Increasing consensus of context-specific metabolic models by integrating data-inferred cell functions. *PLoS computational biology*, 15(4):e1006867, 2019.

[5] Ruth L Seal, Bryony Braschi, Kristian Gray, Tamsin EM Jones, Susan Tweedie, Liora Haim-Vilmovsky, and Elspeth A Bruford. Genenames. org: the hgnc resources in 2023. *Nucleic Acids Research*, 51(D1):D1003–D1009, 2023.

[6] Ines Thiele, Neil Swainston, Ronan MT Fleming, Andreas Hoppe, Swagatika Sahoo, Maike K Aurich, Hulda Haraldsdottir, Monica L Mo, Ottar Rolfsson, Miranda D Stobbe, et al. A community-driven global reconstruction of human metabolism. *Nature biotechnology*, 31(5):419–425, 2013.
