# Supplementary material for: TROPPO: tissue-specific reconstruction and phenotype prediction using omics data
Source: Bioinform Adv. 2025 May 19;5(1):vbaf113. doi: 10.1093/bioadv/vbaf113 (PMC12179386; doi:10.1093/bioadv/vbaf113)
Supplement: vbaf113_Supplementary_Data [file vbaf113_supplementary_data.zip › Supplementary_File_2.pdf]

Supplementary File 2

Omics integration methods: description and benchmark

Alexandre Oliveira[1,*], Jorge Ferreira[1,*], Vítor Vieira[1,*], Bruno Sá[1], Miguel Rocha[1,2,†]

[1]CEB - Centre of Biological Engineering, University of Minho, 4710-057 Braga, Portugal
[2]LABBELS – Associate Laboratory, Braga/Guimarães, Portugal

[†]Corresponding author: mrocha@di.uminho.pt ,
[*]These authors contributed equally for this work.

1. Omics integration methods

Currently, six omics integration methods are implemented in TROPPO. These include Task-driven Integrative Network Inference for Tissues (*tINIT*), Integrative Metabolic Analysis Tool (*iMAT*), Gene Inactivity Moderated by Metabolism and Expression (*GIMME*), *FastCORE*, *SwiftCore*, and Cost Optimisation Reaction Dependency Assessment (*CORDA*). Integration algorithms can be divided into three groups according to the objective of the method: iMAT-like, GIMME-like, and MBA-like.

iMAT-like methods include both *iMAT* and *tINIT*. These methods determine whether a reaction is active or inactive based on the input omics data, ensuring consistency of the context-specific model with the experimental data. These methods can be used for both phenotype prediction and context-specific model reconstruction [6].

GIMME-like methods are phenotype prediction approaches that require the input of required metabolic functions (*RMFs*) in addition to the omics data. *RMFs* are specific metabolic traits that the output model must be able to perform and are enforced by the methods. Examples of such metabolic functions include biomass and ATP production. These methods typically optimise the *RMFs* and then minimise a penalty function to ensure similarity between the predicted values and the experimental data. Even though these methods have been shown to consistently produce more accurate phenotype predictions, selecting *RMFs* is not a straightforward task, particularly in multicellular organisms [6].

In contrast to the previous methods, which predict a flux distribution based on the input data, MBA-like methods only output context-specific models. This group focuses on the categorisation of core and non-core reactions. The core reactions are expected to be active according to the input high-throughput data, while non-core reactions are required to maintain model consistency [6]. These methods include *FastCORE*, *SwiftCore*, and *CORDA*.

1.1. Task-driven Integrative Network Inference for Tissues (*tINIT*)

The *tINIT* [2] algorithm is an extension of the *INIT* algorithm [1], designed to enable the reconstruction of context-specific genome-scale metabolic models (*GSMMs*). Unlike its predecessor, which produced static representations of metabolic networks, *tINIT* incorporates constraints that ensure the reconstructed models can actively perform predefined metabolic tasks. These tasks are user-defined and serve as functional benchmarks for the reconstructed models.

tINIT follows three main steps. Initially, users specify a set of metabolic tasks, and the algorithm identifies the subset of reactions in the reference model that are essential for performing

these tasks. These reactions are forcibly included in the final model to guarantee that the reconstructed model can execute the specified tasks. Instead of simply minimising the number of included reactions, *tINIT* ensures that alternative pathways and isoenzymes are preserved, allowing for biologically relevant flexibility [2].

Once the initial reaction set is determined, the standard *INIT* algorithm [1] is applied with an additional constraint: the identified essential reactions must be retained in the final model. This step ensures that the reconstructed model aligns with the predefined metabolic tasks while also maintaining network consistency. The next stage involves sequentially testing each metabolic task to verify whether it can be performed by the model. If a task fails, gap-filling is applied to introduce the necessary reactions to restore functionality. The sequential nature of this process means that, in theory, task order could influence which reactions are added during gap-filling. However, in practice, this effect is typically minimal due to the robustness of the initial reaction selection process [2].

Beyond task-based reconstruction, *tINIT* introduces two major enhancements over the original *INIT* algorithm. Firstly, it restricts the solution so that reversible reactions cannot carry flux in both directions simultaneously. This constraint prevents the inclusion of non-biological flux loops, thereby improving model reliability. Secondly, *tINIT* provides users with the option to control whether net metabolite production is allowed in the reconstructed model. This feature enhances model flexibility by permitting stricter metabolic constraints when required [2].

1.2. Integrative Metabolic Analysis Tool (*iMAT*)

The *iMAT* algorithm [11] is designed to integrate transcriptomic and proteomic data with *GSMMs* to predict enzyme metabolic fluxes. Unlike experimental techniques that measure fluxes for only a limited number of enzymes, *iMAT* provides a systems-level approach to estimate metabolic states across an entire network. The core idea of *iMAT* is to reconcile gene or protein expression data with a metabolic model using a constraint-based approach, thereby predicting the most likely metabolic flux distribution that is consistent with expression data.

The implementation of *iMAT* begins with the input of gene and/or protein expression data, which must be categorised into discrete expression levels (low, moderate, or high). If continuous data is provided, *iMAT* automatically discretises it. The algorithm then maps this expression information onto the metabolic network, assuming that highly expressed genes are more likely to be associated with active reactions, whereas lowly expressed genes indicate reactions that should have minimal or no flux. By leveraging Mixed-Integer Linear Programming (*MILP*), *iMAT* searches for a flux distribution that maximises consistency with the expression data while maintaining network feasibility. Reactions with indeterminate activity due to alternative pathways or isoenzymes are assigned confidence scores reflecting the likelihood of their activation [11].

1.3. Gene Inactivity Moderated by Metabolism and Expression (*GIMME*)

The *GIMME* algorithm [3] is designed to tailor *GSMMs* to specific cellular phenotypes using gene expression data. The algorithm refines metabolic network reconstructions by integrating transcriptomic data with predefined metabolic objectives, ensuring that the final model remains both biologically relevant and computationally feasible. This method is particularly useful for identifying context-specific metabolic states, as it systematically eliminates reactions associated with lowly expressed genes while preserving essential functionalities.

The implementation of *GIMME* follows a structured approach that requires three key inputs: (1) a genome-scale metabolic reconstruction, (2) quantitative gene expression data, and (3) one or more *RMFs*, such as ATP or biomass production. Initially, reactions associated with genes expressed below a given threshold are marked as inactive. However, since some of these reactions may be essential for achieving the *RMFs*, the algorithm uses linear programming (*LP*) to determine the minimal set of reactions that must be reactivated to maintain metabolic functionality. The algorithm then computes an inconsistency score (*IS*), which quantifies the extent of disagreement

between the gene expression data and the metabolic objective. A lower *IS* indicates a model that is more consistent with the provided expression data [3].

To generate a context-specific metabolic model, *GIMME* proceeds in two main steps. First, it performs Flux Balance Analysis (*FBA*) to determine the maximum achievable flux for the *RMFs* under unconstrained conditions. Next, it applies constraints to enforce a minimum *RMFs* flux (typically set as a percentage of the maximum found in the first step) while optimising for the most consistent reaction set. This ensures that the final network remains functional while aligning as closely as possible with gene expression data. The algorithm assumes a conservative approach to handling missing expression data, treating reactions with no available expression data as active to avoid unwarranted exclusions. The final output consists of a refined metabolic model with the minimal necessary adjustments and a normalised consistency score [3].

1.4. *FastCORE*

The *FastCORE* algorithm [10] is particularly advantageous for large-scale studies where speed and compactness of the resulting models are important considerations. Unlike many other context-specific reconstruction methods that rely on extensive parameter tuning and iterative pruning, *FastCORE* efficiently extracts a flux-consistent subnetwork from a global model while ensuring that a predefined core set of reactions remains active.

The implementation of *FastCORE* begins with a core set of reactions, which represent metabolic activities strongly supported by experimental evidence for a specific cell type or condition. The algorithm then iteratively searches for a minimal network that includes all reactions from this core set while ensuring flux consistency, meaning that each reaction must be capable of carrying nonzero flux in at least one feasible flux distribution. Unlike methods that rely on *MILP* problems, *FastCORE* operates by solving a series of *LP* problems, making it significantly faster and computationally efficient [10].

A key feature of *FastCORE* is its greedy search strategy, which aims to construct the smallest consistent subnetwork. In each iteration, the algorithm identifies a new sparse mode of the global network using L1-norm minimization, ensuring that non-core reactions are added in the most minimal way possible. This differs from traditional pruning-based approaches, such as MBA (Model-Based Approach), which iteratively remove reactions and check for network consistency, a process that is computationally expensive. *FastCORE* instead expands the active reaction set incrementally, ensuring that the final model is both compact and computationally optimal [10].

1.5. *SwiftCore*

SwiftCore [9] is an algorithm designed to efficiently extract a flux-consistent subnetwork from a global model while ensuring the inclusion of a predefined set of core reactions. As a successor to *FastCORE*, *SwiftCore* significantly improves computational efficiency while maintaining the fundamental principles of flux consistency and sparsity in the reconstructed model. The algorithm leverages linear programming (LP) to ensure that all retained reactions are capable of carrying nonzero flux, avoiding blocked reactions in the final network, thereby making it particularly useful for large-scale metabolic network analyses.

SwiftCore follows a greedy approximation strategy to identify the minimal flux-consistent subnetwork containing the specified core reactions. The algorithm starts with a core set of reactions, representing known metabolic activities supported by experimental data. It then iteratively expands this set by incorporating the smallest necessary number of additional reactions to ensure flux consistency, meaning that all included reactions can carry nonzero flux. Unlike pruning-based methods, *SwiftCore* expands the core set by identifying the minimal necessary reactions to ensure a flux-consistent network, reducing computational cost and maintaining functional integrity [9].

A key innovation in *SwiftCore* is its efficient approach to handling reversible reactions. Instead of explicitly assigning reaction directions or solving multiple linear programming (LP) problems for

each reversible reaction (as in *FastCORE*), *SwiftCore* employs a soft preference strategy, allowing the optimisation process to determine the best direction without excessive computational burden. This stochastic component enables exploration of alternative optimum flux distributions, providing more flexibility in selecting a biologically relevant subnetwork [9].

1.6. Cost Optimization Reaction Dependency Assessment (*CORDA*)

Unlike flux-dependent approaches, which generate models based on a single flux distribution, or pruning algorithms, which iteratively remove reactions to create a parsimonious model, *CORDA* [8] introduces a reaction dependency assessment approach. Instead of relying on a fixed flux distribution, *CORDA* systematically evaluates whether a reaction is essential for maintaining network functionality. If a reaction with low confidence is found to be necessary for the model to function, it is included despite its low confidence score.

CORDA classifies reactions into four categories based on experimental evidence: high-confidence (HC), medium-confidence (MC), negative-confidence (NC), and other (OT) reactions. HC reactions are always retained due to their strong experimental support, whereas NC reactions are initially excluded unless they prove essential for maintaining model functionality. The algorithm then performs a dependency assessment, identifying the minimal set of reactions required to sustain metabolic function while ensuring consistency with experimental data. To refine the selection process, *CORDA* introduces a pseudo-metabolite and assigns a cost to each reaction based on its experimental confidence score. This step prioritises reactions with stronger experimental support while allowing for the inclusion of lower-confidence reactions when required [8].

The final model constructed by *CORDA* includes all HC and essential MC reactions while minimising the inclusion of NC reactions. This results in improved agreement with experimental data compared to previous methods like MBA and mCADRE, without compromising model functionality. Additionally, *CORDA*'s dependency assessment provides valuable insights for manual curation, clarifying which reactions are retained due to functional necessity rather than experimental evidence [8]. Compared to previously mentioned approaches, *CORDA* is computationally more efficient, relying solely on *FBA* rather than more complex techniques such as Flux Variability Analysis (*FVA*) or methods that involve *MILP* problems. This efficiency enables *CORDA* to be applied to large-scale tissue-specific reconstructions, making it a powerful tool for studying both healthy and disease-specific metabolic states [8].

2. Omics integration framework benchmark

Initially, performance and computational costs were assessed for all integration methods implemented in TROPPO. To measure peak memory usage during execution, `tracemalloc` was used. The highest amount of RAM consumed (in megabytes, MB) and the execution time (in seconds) was recorded for each process run. Moreover, to evaluate how computational costs scale with larger *GSMMs*, three models were tested: RECON1 [5], redHUMAN RECON2 [7], and RECON3D [4]. Consistent versions of these models were obtained by removing blocked reactions. The model contents are presented in Table 1. Additionally, transcriptomic data from breast cancer cell lines were retrieved from the Cancer Cell Line Encyclopedia (CCLE). Subsequently, transcript activity scores (*TAS*) were calculated, and the ACH-001097 cell line was selected for further analysis.

Table 1. Model details for the three models used in the performance assessment of the integration methods in TROPPO.

Model	Reactions	Metabolites	Genes
RECON1	2467	1586	1478
redHUMAN_RECON2	1396	469	699
RECON3D	10600	5835	2248

For comparing the models reconstructed with TROPPO to those from the original implementations, the redHUMAN RECON2 model was used. Generated models were compared by analysing their reaction content and the number of shared reactions. Moreover, Jaccard distances were calculated following Equation 1:

$$J(A, B) = \frac{|A \cap B|}{|A \cup B|} \tag{1}$$

where, for models A and B, the Jaccard distance is obtained by dividing the number of common reactions (intersection of A and B) by the total number of reactions present in both models (union of A and B).

2.1. Performance and computational costs of each method

Table 2 provides a comparative analysis of execution time and memory usage for all integration methods implemented in TROPPO across three *GSMMs*: RECON1, redHUMAN RECON2, and RECON3D. The results highlight notable differences in computational efficiency among the methods. *FastCORE*, *CORDA*, *GIMME*, and *tINIT* generally exhibit lower execution times and memory consumption compared to *SwiftCore* and *iMAT*, making them more computationally efficient choices, particularly for larger models.

Table 2. Comparison of execution time (s) and memory usage (MB) for all integration methods implemented in TROPPO using Recon1, Recon2, and Recon3D models.

Method	Recon1		Recon2		Recon3D	
	Time (s)	Memory (MB)	Time (s)	Memory (MB)	Time (s)	Memory (MB)
**FastCORE**	97.47	348.85	50.34	114.23	295.42	5065.93
**CORDA**	107.42	356.88	40.95	114.25	1380.46	4909.53
**GIMME**	121.94	286.92	31.69	114.22	270.02	3672.33
**tINIT**	135.42	346.60	40.25	114.23	351.00	4759.66
**Swiftcore**	1180.75	1036.19	542.29	296.13	4652.25	17028.36
**iMAT**	3470.94	962.27	20.51	298.37	*	

* Exceeded available memory using this model.

SwiftCore requires significantly higher computational resources, with execution times exceeding 1000 seconds for RECON1 and reaching over 4600 seconds for RECON3D. RECON3D, being the most complex model, demands substantially more memory across all methods, with *SwiftCore* exceeding 16.6 gigabytes of memory usage. *iMAT* also shows considerable variability, exhibiting an exceptionally high execution time for RECON1 but a notably lower runtime for RECON2. It is also worth noting that, despite the relatively small increase in model size from RECON1 to RECON2, execution time increased by more than 3.6 times. Moreover, for RECON3D, memory usage by *iMAT* exceeded the available computational resources, hence, it was not included in this comparison. These findings underscore the trade-offs between computational efficiency and method complexity, emphasising the importance of selecting an appropriate integration method based on the scale of the template model and available resources.

2.2. Comparing TROPPO's integration methods with the original implementation

Context-specific models were generated using all methods implemented in TROPPO, as well as their original implementation. The MATLAB implementations available through COBRA toolbox were used for all algorithms, except for CORDA, which is available in Python. Concerning the comparison between integration algorithms, Jaccard distances and reaction contents of models

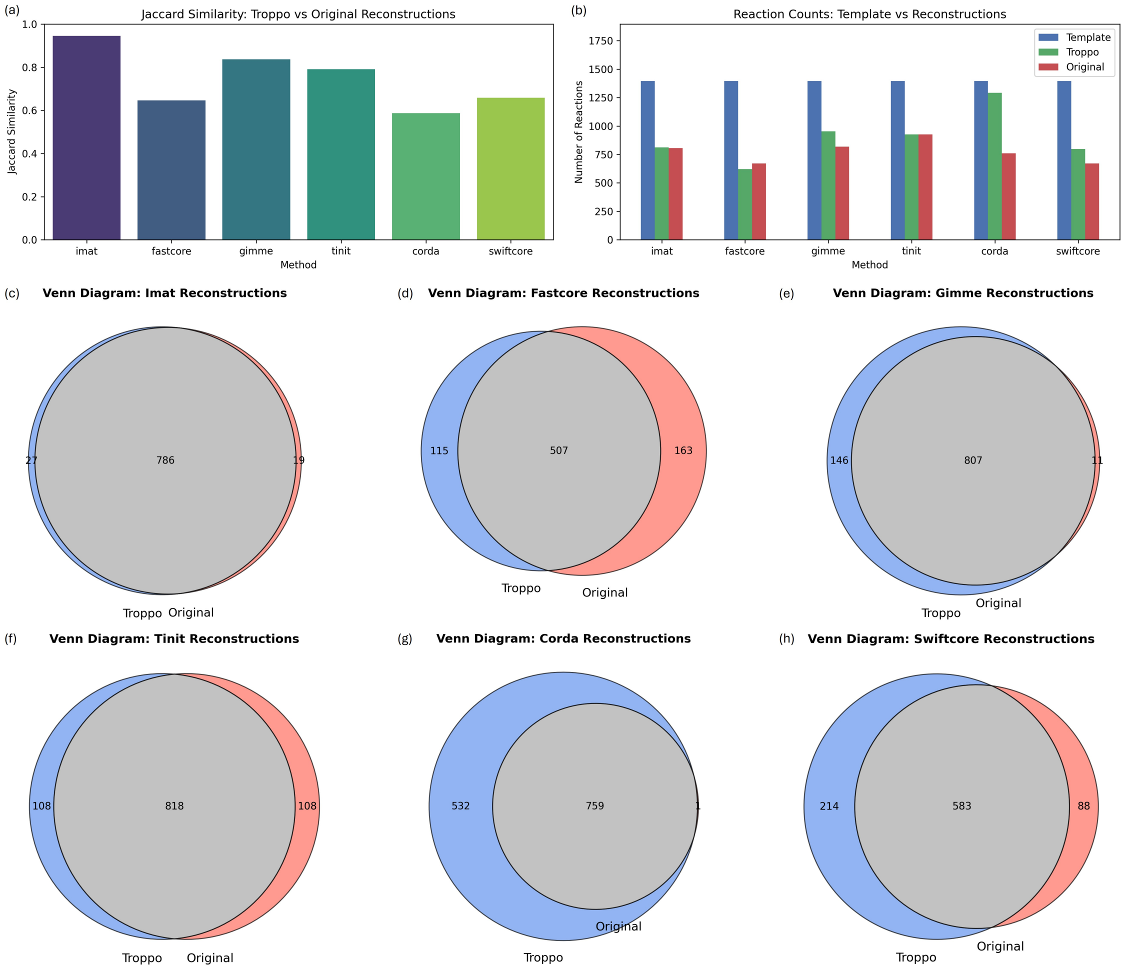

Figure 1. Benchmark results from the omics integration methods implemented in TROPPO. Jaccard distances were calculated between the model generated with the original algorithm implementation and TROPPO (a). The number of reactions was also compared between both models to assess how they differed (b). Venn diagrams demonstrating reaction space intersection for *iMAT* (c), *FastCORE* (d), *GIMME* (e), *tINIT* (f), *CORDA* (g), and *SwiftCore* (h) are also shown.

generated with TROPPO and the original algorithm implementations were analysed (Figure 1). Regarding Jaccard distances, the methods that produced models most similar to the original implementations were *iMAT*, *GIMME*, and *tINIT*. This is further confirmed by examining reaction contents, where all methods generated models closely resembling the original implementations, matching the majority of reactions in the context-specific models.

Moreover, with similar Jaccard distances, *FastCORE* and *SwiftCore* produced models comparable to the original implementations. However, when using *FastCORE*, a high number of reactions from the original method were deleted in the models generated by TROPPO. If this method is chosen for generating context-specific models, despite being the fastest option, this trade-off should be taken into consideration. Similarly, *SwiftCore* introduced a considerable number of reactions that were not present in the models produced by the original implementation, which should also be kept in mind.

On the other hand, *CORDA* yielded the model with the lowest Jaccard distance. Even though TROPPO's model included most of the reactions that the original algorithm's model had, this happened because very few reactions were removed from the original template model. This should be taken into account before using this method, as the original implementation should be preferred. Hence, *CORDA* presents a challenge that should be addressed in the future to improve the

framework.

References

[1] Rasmus Agren, Sergio Bordel, Adil Mardinoglu, Natapol Pornputtapong, Intawat Nookaew, and Jens Nielsen. Reconstruction of Genome-Scale Active Metabolic Networks for 69 Human Cell Types and 16 Cancer Types Using INIT. *PLoS Computational Biology*, 8(5):e1002518, 2012.

[2] Rasmus Agren, Adil Mardinoglu, Anna Asplund, Caroline Kampf, Mathias Uhlen, and Jens Nielsen. Identification of anticancer drugs for hepatocellular carcinoma through personalized genome-scale metabolic modeling. *Molecular Systems Biology*, 10(3):721, 2014.

[3] Scott A Becker and Bernhard O Palsson. Context-specific metabolic networks are consistent with experiments. *PLoS computational biology*, 4(5):e1000082, 2008.

[4] Elizabeth Brunk, Swagatika Sahoo, Daniel C Zielinski, Ali Altunkaya, Andreas Dräger, Nathan Mih, Francesco Gatto, Avlant Nilsson, German Andres Preciat Gonzalez, Maike Kathrin Aurich, Andreas Prlić, Anand Sastry, Anna D Danielsdottir, Almut Heinken, Alberto Noronha, Peter W Rose, Stephen K Burley, Ronan M T Fleming, Jens Nielsen, Ines Thiele, and Bernhard O Palsson. Recon3D enables a three-dimensional view of gene variation in human metabolism. *Nature Biotechnology*, 36(3):272–281, 2018.

[5] Natalie C Duarte, Scott A Becker, Neema Jamshidi, Ines Thiele, Monica L Mo, Thuy D Vo, Rohith Srivas, and Bernhard Ø Palsson. Global reconstruction of the human metabolic network based on genomic and bibliomic data. *Proceedings of the National Academy of Sciences*, 104(6):1777–1782, 2007.

[6] Semidán Robaina Estévez and Zoran Nikoloski. Generalized framework for context-specific metabolic model extraction methods. *Frontiers in Plant Science*, 5(September):1–11, 2014.

[7] Maria Masid, Meric Ataman, and Vassily Hatzimanikatis. Analysis of human metabolism by reducing the complexity of the genome-scale models using redhuman. *Nature communications*, 11(1):2821, 2020.

[8] André Schultz and Amina A Qutub. Reconstruction of tissue-specific metabolic networks using corda. *PLoS computational biology*, 12(3):e1004808, 2016.

[9] Mojtaba Tefagh and Stephen P Boyd. Swiftcore: a tool for the context-specific reconstruction of genome-scale metabolic networks. *BMC bioinformatics*, 21:1–14, 2020.

[10] Nikos Vlassis, Maria Pires Pacheco, and Thomas Sauter. Fast reconstruction of compact context-specific metabolic network models. *PLoS computational biology*, 10(1):e1003424, 2014.

[11] Hadas Zur, Eytan Ruppin, and Tomer Shlomi. imat: an integrative metabolic analysis tool. *Bioinformatics*, 26(24):3140–3142, 2010.
