# Supplementary material for: TROPPO: tissue-specific reconstruction and phenotype prediction using omics data
Source: Bioinform Adv. 2025 May 19;5(1):vbaf113. doi: 10.1093/bioadv/vbaf113 (PMC12179386; doi:10.1093/bioadv/vbaf113)
Supplement: vbaf113_Supplementary_Data [file vbaf113_supplementary_data.zip › Supplementary_File_3.pdf]

Supplementary File 3

A pipeline for large-scale reconstruction and validation of context-specific models

Alexandre Oliveira[1,*], Jorge Ferreira[1,*], Vítor Vieira[1,*], Bruno Sá[1], Miguel Rocha[1,2,†]

[1]CEB - Centre of Biological Engineering, University of Minho, 4710-057 Braga, Portugal
[2]LABBELS – Associate Laboratory, Braga/Guimarães, Portugal

[†]Corresponding author: mrocha@di.uminho.pt ,
[*]These authors contributed equally for this work.

This case study examines the pipeline used by [6] for large-scale context-specific model reconstruction. The authors employed this approach to reconstruct over 6,000 models for 733 cell lines from the Cancer Cell Line Encyclopedia (CCLE) database [3]. Their methodology was validated using an MCF7 cell line sample [4, 2], generating 320 models through various algorithms and parameter combinations. Furthermore, the autors also evaluated which models best aligned with experimentally determined essential genes and fluxomics data. For simplicity, this example will focus only on the parameter combination that yielded the best-performing models.

In this notebook, the focus will be the reconstruction of context-specific models for the MCF7 cell line using the Human-GEM model [5] as a template. The dataset was obtained from all 733 CCLE samples to calculate transcript activity scores (TASs) for each of the 80 possible combinations of the Global (5 combinations), Local T1 (25 combinations), and Local T2 (50 combinations) strategies. From these, we selected the combinations specific to the MCF7 cell line (ACH-000019). Each combination was reconstructed using either a min/max or min/sum function to calculate scores for each reaction with FastCORE [7] or tINIT [1], resulting in 320 context-specific models. These models were further refined using EFM gap-fill and validated.

The jupyter notebook and all files required for this analysis are available for download on *Troppo's* GitHub page (`https://github.com/BioSystemsUM/troppo/tree/master/examples/mcf7_case_study`).

1. Pipeline Overview

The pipeline employed for this case study will follow these steps:

1. **Context-Specific Model Reconstruction**: Reconstruct 320 context-specific models for the MCF7 cell line using all possible scoring combinations with both FastCORE and tINIT algorithms.

2. **Fine-Tune Models with Gap-Filling**: Apply gap-filling for growth under open medium conditions using the `EFMGapfill` method.

3. **Task Evaluation**: Evaluate the performance of metabolic tasks for the fine-tuned models.

2. Context-Specific Model Reconstruction

2.1. Required Imports and Define Paths

```
import os
import pandas as pd
import re

from cobra.io import read_sbml_model
from cobamp.utilities.parallel import batch_run
from troppo.methods_wrappers import ReconstructionWrapper
from troppo.omics.core import TypedOmicsMeasurementSet,
    IdentifierMapping
from itertools import chain

DATA_PATH = 'data/CCLE_expression_ACH-000019_scores.csv'
CS_MODEL_DF_FOLDER = 'results/reconstructions/mcf7_comparison'
CS_MODEL_NAMES = 'cs_models_all_combinations'
GENE_NAME_GRAB_PATTERN = 'ENSG[0-9]*'
PROTECTED_REACTION_LIST = 'biomass_human,HMR_10023,HMR_10024'
ALGORITHM_OPTIONS = 'fastcore,tinit'
INTFUNCTION_OPTIONS = 'minsum,minmax'
INDEX_COLUMNS = '0,1,2,3'
NTHREADS = 12
CS_MODEL_DF_FILE = os.path.join(CS_MODEL_DF_FOLDER, CS_MODEL_NAMES
    + '.csv')

if not os.path.exists(CS_MODEL_DF_FOLDER):
    os.makedirs(CS_MODEL_DF_FOLDER)
```

2.2. Read and Process the Omics Dataset

For this case study, the input will be the TASs for the ACH-000019 sample of the CCLE dataset. TAS were previously calculated using the `GeneLevelThresholding` class for all possible combinations of thresholding strategies.

```
# This is needed only if you need to convert genes from one
    nomenclature to another
mapping = IdentifierMapping('human_transcriptomics',
        pd.read_csv('http://ftp.ebi.ac.uk/pub/databases/genenames/'
        'out_of_date_hgnc/tsv/hgnc_complete_set.txt',
        index_col=0, sep='\t'))

exp_data = pd.read_csv(DATA_PATH, index_col=list(map(int,
    INDEX_COLUMNS.split(','))))
exp_data = exp_data.tail(2)
exp_data
```

Load the dataset into a `OmicsMeasurmentSet` object with the dataframe components.

```
omics_mset = TypedOmicsMeasurementSet(exp_data.index,
    exp_data.columns, exp_data.values, mapping)
```

The gene identifiers on the dataset have two different nomenclatures, HGNC Symbol and Ensembl Gene ID. Since the model that is going to be used the Ensembl Gene IDs, the column names must be changed.

```
if GENE_NAME_GRAB_PATTERN != '':
    ensembl_patt = re.compile(GENE_NAME_GRAB_PATTERN)
    omics_mset.column_names = [ensembl_patt.findall(k)[0] for k in
        omics_mset.column_names]
```

After converting the gene IDs to the same nomenclature as the genes in the model, the data will be converted to a dictionary to simplify the process of generating all possible combinations to be used for each algorithm on the reconstruction process.

```
data_dicts = {'_'.join(map(str, k)): v for k, v in
    omics_mset.data.T.to_dict().items()}
```

The biomass reaction will also be marked as a protected reaction so it doesn't get removed from the core.

```
protected = list(map(lambda x: x.strip(),
    PROTECTED_REACTION_LIST.split(',')))
```

Generate all possible combinations

To use all possible combinations, each of the thresholding strategies need to be tested using different and/or functions during the `ScoreIntegration` process. These are associated with how the score is attributed to reactions according to its Gene-Protein rule (GPRs), more specifically:

- `and_func`: a function that is used to combine the scores of the genes associated with a reaction for AND rules in the GPR. Typically, the minimum function is used. This means that the score of a reaction with AND in its GPRs will be the minimum score of the genes associated with it.
- `or_func`: a function that is used to combine the scores of the genes associated with a reaction for OR rules in the GPR. Typically, sum or maximum functions can be used. If the `sum` function is used, the score of a reaction with OR in its GPRs will be the sum of the scores of the genes associated with it. If `max` is used, the score for the gene with the maximum score will be used.

Hence, the following step is to generate a dictionary with all combinations of parameters plus and/or functions.

```
avaliable_funcs = list(map(lambda x: x.strip(),
    INTFUNCTION_OPTIONS.split(',')))
funcs = {k: v for k, v in {'minsum': (min, sum),
                           'minmax': (min, max)}.items() if k in
                               avaliable_funcs}
runs = dict(chain(*[[((k, n), (aof, v)) for k, v in
    data_dicts.items()] for n, aof in funcs.items()]))
```

2.3. Load the HumanGEM Model

For this case study, a simplified version of the generic Human-GEM model was used. The changes performed to the model were the following:

1. Remove Boundary Metabolites: Eliminate boundary metabolites from the model.
2. Remove Blocked Reactions: Exclude reactions that are blocked and do not contribute to the metabolic network.
3. Adjust Lower Bound of `HMR_10024` Reaction: Set the lower bound of the `HMR_10024` reaction to zero. This reaction represents an exchange reaction for biomass and is necessary to close it to ensure that biomass is not entering the system.

```
model = read_sbml_model('data/Human-GEM.xml')
model

model.reactions.get_by_id('HMR_10024').bounds = (0, 1000)
```

Create a model wrapper

The `ModelBasedWrapper` class is used to wrap the model so that it can be used by *Troppo*.

Relevant arguments from this class include:

- `model`: the model to be wrapped.
- `ttg_ratio`: the ratio between the number of reactions to be selected and the total number of reactions in the model.
- `gpr_gene_parse_function`: a function that parses the GPRs of the model. This is used to map the identifiers in the omics data to the identifiers in the model.

Important attributes from this class include:

- `model_reader`: a COBRAModelObjectReader instance containing all the information of the model, such as reaction_ids, metabolite_ids, GPRs, bounds, etc.
- `S`: the stoichiometric matrix of the model.
- `lb`: the lower bounds of the reactions in the model.
- `ub`: the upper bounds of the reactions in the model.

In this specific example, we will use the `ReconstructionWrapper` class instead of the base `ModelBasedWrapper` class.

```
rw = ReconstructionWrapper(model, ttg_ratio=9999)
```

2.4. Model Reconstruction

The reconstruction process will be carried out through batch functions for a more time-efficient process. To achieve this, it is first necessary to define the functions that will be parallelized. These functions must be specific to the reconstruction algorithm being used and follow a specific structure:

- Convert the omics data into an `OmicsContainer` object.
- Define a custom `ScoreIntegration` function.
- Apply `run_from_omics` method from the `ReconstructionWrapper` object.

Parameters required for the `run_from_omics` function:

- `omics_data`: The omics data container for the sample.
- `algorithm`: A string specifying the reconstruction algorithm.
- `and_or_funcs`: A tuple containing functions for the AND and OR operations of the GPR.
- `integration_strategy`: A tuple with the integration strategy and the function to apply to the scores.
- `solver`: The solver for optimization.
- `**kwargs**`: Additional parameters specific to the algorithm used.

Output: The `run_from_omics` method returns a dictionary with all model reactions and their respective Boolean values, indicating whether each reaction is active in the model. Note that this function will run for each sample individually, so the dictionary will only contain results for one sample at a time.

```
def fastcore_reconstruction_func(score_tuple, params):
    from troppo.omics.core import OmicsContainer
    aofx, data_dict = score_tuple
    oc_sample = OmicsContainer(omicstype='transcriptomics',
       condition='x', data=data_dict, nomenclature='custom')
    r_wrapper = [params[k] for k in ['rw']][0]  # load parameters
    protected_core = params['protected']
    t = 0

    def integration_fx(data_map):
        return [[k for k, v in data_map.get_scores().items() if
                  (v is not None and v > t) or k in protected_core]]

    try:
        return r_wrapper.run_from_omics(omics_data=oc_sample,
            algorithm='fastcore', and_or_funcs=aofx,
                  integration_strategy=('custom', [integration_fx]),
                      solver='CPLEX')
    except Exception as e:
        print(e)
        return {r: False for r in r_wrapper.model_reader.r_ids}


def tinit_reconstruction_func(score_tuple, params):
    from troppo.omics.core import OmicsContainer
    aofx, data_dict = score_tuple
```

```
    oc_sample = OmicsContainer(omicstype='transcriptomics',
        condition='x', data=data_dict, nomenclature='custom')
    r_wrapper = [params[k] for k in ['rw']][0]  # load parameters
    protected_core = params['protected']
    try:
        def tinit_integration_fx(data_map):
            maxv = max([k for k in data_map.get_scores().values()
                if k is not None])
            scores = {k: (v / maxv if v < 0 else v) if v is not
                None else 0 for k, v in
                data_map.get_scores().items()}
            scores.update({x: max(scores.values()) for x in
                protected_core})
            return scores

        return r_wrapper.run_from_omics(omics_data=oc_sample,
            algorithm='tinit', and_or_funcs=aofx,
             integration_strategy=('custom',
                [tinit_integration_fx]), solver='CPLEX')
    except Exception as e:
        print(e)
        return {r: False for r in r_wrapper.model_reader.r_ids}


% func_map = {'tinit': tinit_reconstruction_func, 'fastcore':
    fastcore_reconstruction_func}
func_map = {'tinit': tinit_reconstruction_func}
safe_threads = {'tinit': 1, 'fastcore': 2}
result_dicts = {}
```

Define the `batch_reconstruct` function to parse the configurations and run the `batch_run` method from COBAMP.

For this function, the required parameters are:

- `function`: The function to be parallelized.
- `sequence`: The sequence where the parallelized function is to be applied.
- `paramargs`: The specific parameters of the function.
- `threads`: The number of threads to use for parallelization.

```
def batch_reconstruct(runs_config, rec_wrapper, core, rec_func,
    algo_name, max_threads=NTHREADS):
    labs, iters = zip(*runs_config.items())
    tlabs = [tuple([algo_name] + list(lab)) for lab in labs]
    output = batch_run(rec_func, iters, {'rw': rec_wrapper,
        'protected': core}, threads=min(len(runs_config),
        max_threads))
    return dict(zip(tlabs, output))
```

The final step is to call the `batch_reconstruct` function for each algorithm and save the results.

```
avaliable_algos = list(map(lambda x: x.strip(),
    ALGORITHM_OPTIONS.split(',')))
for algname, func in func_map.items():
     if algname in avaliable_algos:
          result_dicts.update(batch_reconstruct(runs, rw, protected,
              func, algname, safe_threads[algname]))
```

3. Gap-fill for growth under open medium conditions

3.1. Imports and path definition

```
from cobamp.wrappers.external_wrappers import get_model_reader
from troppo.methods_wrappers import GapfillWrapper
from troppo.methods.gapfill.efm import EFMGapfillProperties

MODEL_DF_PATH_BIOMASS_GAPFILL = os.path.join(CS_MODEL_DF_FOLDER,
    CS_MODEL_NAMES + '_biomass.csv')

model = read_sbml_model('data/Human-GEM.xml')
model
```

For this case study, gap-filling was performed to ensure that biomass production was achieved for all models under open medium conditions. The first step in this process was to create a `ConstraintBasedModel` object from CoBAMP using the `to_cobamp_cbm()` method.

```
cobamp_model = get_model_reader(model).to_cobamp_cbm('CPLEX')
```

3.2. EFM Gapfilling

To use a COBRA model object for gap-filling with TROPPO, a `ModelBasedWrapper` object is required. For this specific task, we will use the `GapfillWrapper` class instead of the base `ReconstructionWrapper` class used in the previous step.

```
gw = GapfillWrapper(model)
```

Next, all boundary reactions are retrieved to ensure they will be part of the final reactions of the fine-tuned models.

```
exchanges =
    set(chain(*cobamp_model.get_boundary_reactions().values()))
gapfill_results = {}
```

For gap-filling, the following methods are required for each model:

1. **Simulate the Reconstructed Model**: Check if biomass production is achieved. If not, perform gap-filling.
2. **Run the EFM Algorithm**: Use the EFM algorithm to perform gap-filling.

3. **Update Reconstruction Results**: Incorporate the reactions required to achieve biomass production into the set of reconstruction results.

This method can be further adapted to ensure biomass production under specific medium conditions.

```
for i, row in result_df.T.items():
    if i not in gapfill_results.keys():
        activity = row.to_dict()

        for k in exchanges:
            activity[k] = True

        inactive_rxs = set([k for k, v in activity.items() if not
           v])

        with cobamp_model as m:
            for rx in inactive_rxs:
                m.set_reaction_bounds(rx, lb=0, ub=0)
            sol = m.optimize({'biomass_human': 1}, False)

        if sol.status() != 'optimal' or sol.objective_value() <
           1e-3:
            print('Model', i, 'did not pass:', sol)
            gapfill_sol = gw.run(avbl_fluxes=list(inactive_rxs),
                                 algorithm='efm',
                                 ls_override={'produced':
                                    ['temp001c']})
            for k in gapfill_sol[0]:
                activity[k] = True
            with cobamp_model as m:
                for rx in [k for k, v in activity.items() if not
                   v]:
                    m.set_reaction_bounds(rx, lb=0, ub=0)
                gsol = m.optimize({'biomass_human': 1}, False)
                print('Solution after gapfilling:', gsol)
        else:
            print('Model', i, 'passed:', sol)
        gapfill_results[i] = activity
```

4. Evaluate Essential Tasks

4.1. Imports and Paths

```
from troppo.tasks.core import TaskEvaluator
from troppo.tasks.task_io import ExcelTaskIO
from cobamp.utilities.file_io import pickle_object
import numpy as np

TASK_FILE_PATH = 'data/metabolicTasks_Essential.xlsx'
CS_MODEL_SET_NAME = 'cs_models_all_combinations_biomass'
TASK_RESULTS_FOLDER = os.path.join(CS_MODEL_DF_FOLDER,
   'task_evaluation')
```

```
TASK_RESULTS_PATH = os.path.join(TASK_RESULTS_FOLDER,
    CS_MODEL_SET_NAME+'_taskeval.pkl')
TASK_RESULTS_DF = os.path.join(TASK_RESULTS_FOLDER,
    CS_MODEL_SET_NAME+'_taskeval.csv')

if not os.path.exists(TASK_RESULTS_FOLDER):
    os.makedirs(TASK_RESULTS_FOLDER)

AVAILABLE_THREADS = 8
```

4.2. Load metabolic tasks

The first step of the Task evaluation portion of the pipeline is to load a file with the metabolic tasks for the Human-GEM model. This file can be found on TROPPO's GitHub page and will be loaded with the `ExcelTaskIO` subclass. Since the tasks file is a `.xlsx` file, it is possible that an `xlrd` dependency is required. If that is the case, use `pip` to install the dependency into your environment.

```
task_list = ExcelTaskIO().read_task(TASK_FILE_PATH)
```

Further processing of the task list to account for changes made to the model:

```
metab_map = {m.name + '[' + m.compartment + ']': m.id if
    m.compartment != 'x' else m.id[:-1] + 's' for m in
                       model.metabolites}
metab_map['NEFA blood pool in[x]'] = 'm02560s'
replace_func = lambda x: metab_map[x] if x in metab_map.keys()
    else x

for t in task_list: t.id_replace(replace_func)  # only works for
    inflow_dict/outflow_dict
for t in task_list:
     t.reaction_dict = {k: [{metab_map[i]: j for i, j in
         v[0].items() if i in metab_map.keys()}, v[1]]
                              for k, v in t.reaction_dict.items()}

task_list[27].outflow_dict.update(task_list[27].inflow_dict)
del task_list[27].outflow_dict['ALLMETSIN[s]']
```

4.3. Task evaluation

In order to perform the task evaluation we need to create a copy of the model to avoid making changes to the template model. Subsequently, boundary reactions are to be removed and drains set to zero.

```
task_model = model.copy()

for rx in task_model.boundary: rx.bounds = (0, 0)

task_model_no_boundary = task_model.copy()
```

```
task_model_no_boundary.remove_reactions(
               task_model_no_boundary.boundary)
task_model_no_boundary.remove_reactions([k for k in
    task_model_no_boundary.reactions if len(k.metabolites) == 0])
```

The class responsible for task evaluation in TROPPO is called `TaskEvaluator`. This class serves as a wrapper around a model, allowing for the evaluation of specific tasks on the model. It can be used to evaluate either a single task or a batch of tasks on a batch of models. To initialize a `TaskEvaluator` object, you need to pass a model object and the tasks to evaluate. The tasks should be instances of the `Task` class, which is a simple data structure containing the following fields:

- `reaction_dict`: A dictionary with reaction identifiers as keys and a dictionary with metabolites and their respective stoichiometry as values. For example: rxd = 'r1':('m1': -1, 'm2': 2, (lb, ub)), ...
- `inflow_dict`: A dictionary with metabolite identifiers as keys and the inflow rate as values. For example: inflow = 'm1': (1, 1), ...
- `outflow_dict`: A dictionary with metabolite identifiers as keys and the outflow rate as values. For example: outflow = 'm5': (5, 5), ...

This structure allows you to define and evaluate the metabolic tasks on your model efficiently.

```
tev = TaskEvaluator(model=task_model_no_boundary, tasks=task_list)
```

The following lines represent the final step in the pipeline, where task evaluation is performed:

1. **Split Data and Iterate**: The script splits the `biomass_gapfill_models` DataFrame into chunks based on the number of available threads (`AVAILABLE_THREADS`). It then iterates over these chunks.
2. **Prepare Data for Evaluation**: Within each chunk, it prepares a list of boundary changes. Each change sets the lower and upper bounds of certain reactions to (0, 0) if the reaction value is not present in the data.
3. **Evaluate Tasks**: The script evaluates the tasks in parallel using the `tev.batch_evaluate` method.
4. **Update Results**: The evaluation results are updated in the `task_eval_results` dictionary. This dictionary is indexed by the original DataFrame indices, with each entry containing the evaluation results.

This step ensures that all evaluated tasks are properly stored and managed after processing, completing the pipeline workflow.

```
task_eval_results = {}

for i, sub_model_df in
    enumerate(np.array_split(biomass_gapfill_models[[r.id for r in
    task_model_no_boundary.reactions]],
                                        AVAILABLE_THREADS)):
     print('Model chunk #'+str(i+1))
     bound_changes = [{k:(0, 0) for k,v in row.to_dict().items() if
         not v}
```

```
                        for i, row in sub_model_df.iterrows()]
    task_eval_results.update({sub_model_df.index[k]: {i:j[0] for
       i,j in v.items()}
                                  for k,v in
                                     tev.batch_evaluate(bound_changes,
                                     AVAILABLE_THREADS).items()})
    pickle_object(task_eval_results,TASK_RESULTS_PATH+'.'+str(i))
```

The results will be saved in a pickle file. Nevertheless, we can convert the resulting dictionary into a pandas DataFrame to assess the results.

```
tevr = pd.DataFrame(task_eval_results).T
tevr

tevr.to_csv(TASK_RESULTS_DF)
```

5. Conclusion

In this study, we present a comprehensive pipeline for the large-scale reconstruction and validation of context-specific metabolic models, exemplified by the MCF7 cell line. Our approach integrates transcriptomic data from CCLE with metabolic modeling frameworks, leveraging multiple reconstruction algorithms (FastCORE and tINIT) and a gap-filling strategy to ensure biologically relevant predictions. By evaluating essential metabolic tasks, we demonstrated the accuracy and reliability of the reconstructed models in capturing key metabolic functions. This pipeline provides a scalable framework for studying metabolism in diverse cellular contexts, facilitating insights into metabolic reprogramming in human diseases. The implementation and datasets used in this study are publicly available, ensuring reproducibility and further applications. For further details on the analyses performed, we recommend consulting the original publication [6].

References

[1] Rasmus Agren, Adil Mardinoglu, Anna Asplund, Caroline Kampf, Mathias Uhlen, and Jens Nielsen. Identification of anticancer drugs for hepatocellular carcinoma through personalized genome-scale metabolic modeling. *Molecular systems biology*, 10(3):721, 2014.

[2] Joshua M Dempster, Jordan Rossen, Mariya Kazachkova, Joshua Pan, Guillaume Kugener, David E Root, and Aviad Tsherniak. Extracting biological insights from the project achilles genome-scale crispr screens in cancer cell lines. *BioRxiv*, page 720243, 2019.

[3] Mahmoud Ghandi, Franklin W Huang, Judit Jané-Valbuena, Gregory V Kryukov, Christopher C Lo, E Robert McDonald III, Jordi Barretina, Ellen T Gelfand, Craig M Bielski, Haoxin Li, et al. Next-generation characterization of the cancer cell line encyclopedia. *Nature*, 569(7757):503–508, 2019.

[4] Robin M Meyers, Jordan G Bryan, James M McFarland, Barbara A Weir, Ann E Sizemore, Han Xu, Neekesh V Dharia, Phillip G Montgomery, Glenn S Cowley, Sasha Pantel, et al. Computational correction of copy number effect improves specificity of crispr–cas9 essentiality screens in cancer cells. *Nature genetics*, 49(12):1779–1784, 2017.

[5] Jonathan L Robinson, Pınar Kocabaş, Hao Wang, Pierre-Etienne Cholley, Daniel Cook, Avlant Nilsson, Mihail Anton, Raphael Ferreira, Iván Domenzain, Virinchi Billa, et al. An atlas of human metabolism. *Science signaling*, 13(624):eaaz1482, 2020.

[6] Vítor Vieira, Jorge Ferreira, and Miguel Rocha. A pipeline for the reconstruction and evaluation of context-specific human metabolic models at a large-scale. *PLoS computational biology*, 18(6):e1009294, 2022.

[7] Nikos Vlassis, Maria Pires Pacheco, and Thomas Sauter. Fast reconstruction of compact context-specific metabolic network models. *PLoS computational biology*, 10(1):e1003424, 2014.
