# Supplementary material for: TROPPO: tissue-specific reconstruction and phenotype prediction using omics data
Source: Bioinform Adv. 2025 May 19;5(1):vbaf113. doi: 10.1093/bioadv/vbaf113 (PMC12179386; doi:10.1093/bioadv/vbaf113)
Supplement: vbaf113_Supplementary_Data [file vbaf113_supplementary_data.zip › Supplementary_File_4.pdf]

Supplementary File 4

A pipeline for context-specific model Reconstruction in Healthy and COVID-19 infected tissues

Alexandre Oliveira[1,*], Jorge Ferreira[1,*], Vítor Vieira[1,*], Bruno Sá[1], Miguel Rocha[1,2,†]

[1]CEB - Centre of Biological Engineering, University of Minho, 4710-057 Braga, Portugal
[2]LABBELS – Associate Laboratory, Braga/Guimarães, Portugal

[†]Corresponding author: mrocha@di.uminho.pt ,
[*]These authors contributed equally for this work.

1. COVID-19 Case Study

The goal of this case study is to explore metabolic interactions between SARS-CoV-2 and its host during infection. To achieve this, bulk RNA-seq data will be used to reconstruct context-specific models of several healthy and infected tissues. The transcriptomics data will be integrated into the Human-GEM model [4] using the FastCORE algorithm [6].

The dataset selected for this case study is comprised of two distinct datasets:

1. **GEO: GSE150316**: This dataset is from the study titled *"Temporal and spatial heterogeneity of host response to SARS-CoV-2 pulmonary infection"* by Desai and colleagues [1]. It includes analyses of autopsy specimens from 24 COVID-19 patients, with samples collected from various tissues. However, this study lacks healthy samples for contrast with the infected tissues.
2. **Genotype-Tissue Expression (GTEx) Portal**: To supplement the lack of healthy samples, data from the GTEx Portal [5] was used. This dataset includes data from several non-diseased tissues.

Given that raw count values were available for the **GSE150316** dataset, the solution was to apply the same normalisation pipeline used for the GTEx data. Specifically, median expression values for all genes were calculated, followed by transcripts per million (TPM) normalisation, with this process repeated for each tissue. The intersection of tissues found in both datasets was used, resulting in five different tissues: lung, heart, kidney, liver, and intestine. This dataset and model used here are available for download on *Troppo's* GitHub page (`https://github.com/BioSystemsUM/troppo/tree/master/examples/covid19_case_study`).

2. Pipeline Overview

The pipeline employed for this case study will follow these steps:

1. **Initial Setup**: Load the dataset and model.
2. **Gene-Level Thresholding**: Calculate Transcript Activity Scores (TAS) using the `GeneLevelThresholding` class.
3. **Run FastCORE**: Use *Troppo's* FastCORE algorithm for multiple samples with the `run_from_omics` function of the `ReconstructionWrapper` class.

2.1. Initial Setup

Required Imports

```python
import re
import pandas as pd
import seaborn as sns
import matplotlib.pyplot as plt
from cobra.io import read_sbml_model
from troppo.omics.readers.generic import TabularReader
from troppo.methods_wrappers import ReconstructionWrapper
from cobamp.utilities.parallel import batch_run
from troppo.omics import GeneLevelThresholding
```

Load the model

```python
model = read_sbml_model('HumanGEM_Consistent_COVID19_HAM.xml')
model
```

Note that for this study we are using a modified version of the HumanGEM model. Three main changes were performed:

- **Environmental Conditions**: The model's environmental conditions were altered to represent a HAM medium.
- **Viral Biomass Objective Function**: Added a viral biomass objective function to model SARS-CoV-2 replication.
- **Create a Consistent Model**: Blocked reactions were removed to create a consistent model. This step simplified the model and ensured consistency, as required by the *FastCORE* algorithm. Blocked reactions were identified using the `find_blocked_reactions` function from COBRApy, which utilizes *Flux Variability Analysis (FVA)* to detect reactions with no flux. After identifying the blocked reactions, they were removed, resulting in a consistent model.

Load the omics data

```python
omics_data = pd.read_csv(filepath_or_buffer='Desai-GTEx_ensembl.csv', index_col=0)
omics_data

omics_data.T.describe()
```

Note that in *Troppo*, omics datasets must have samples as rows and gene IDs as columns.

2.2. Gene-Level Thresholding

However, other strategies were introduced that use thresholds to assess which genes are active before the integration process by calculating their TAS. In *Troppo*, such methods can be used through the `GeneLevelThresholding` class.

This class is used to transform the dataframe containing the omics data and perform gene-level thresholding on omics data. It currently supports Global and Local thresholding approaches described by Richelle, Joshi, and Lewis (2019) [3]. These include:

- **Global**: Genes with a value greater than the lower global threshold (GTL) are considered active, and below inactive.

- **Local T1**: Genes with a value lower than the GTL are considered inactive; for genes with a value greater than the GTL, if the value is lower than the local threshold (LT), the gene is considered inactive; otherwise, it is considered active.
- **Local T2**: Genes with a value lower than the GTL are considered inactive; genes with a value greater than the upper global threshold (GTU) are considered active; for genes with a value between the GTL and the GTU, they are only considered active if their value is greater than the LT.

Thresholds are selected in accordance with the distribution of the data. The numbers in the thresholding options represent the position of the value to use. Currently, the options are: `[0.1, 0.25, 0.5, 0.75, 0.9]`; the threshold value will then be the value in the dataset that corresponds to that quantile.

To assess which thresholds to select, it is wise to first look at the data distribution and the values of these thresholds.

```
fig, axs = plt.subplots(1, 1, figsize=(5, 5))
sns.histplot(omics_data.values.flatten(),ax=axs)

for quantile in [0.25, 0.5, 0.75, 0.9]:
    quantile_value = pd.Series(omics_data.values.flatten()).quantile(quantile)
    axs.axvline(quantile_value, color='red', linestyle='--')
    axs.text(quantile_value + 0.3, 36000, f'{quantile} ', rotation=90,
        verticalalignment='center', horizontalalignment='left', color='red')
```

For this example, the **Local T2** approach will be used. While literature suggests using the 25th and 75th percentiles for global thresholds, the 25th percentile in this case is zero. Therefore, the 50th percentile will be chosen for the global threshold lower. For the local threshold, the 50th percentile will also be used.

To instantiate the `GeneLevelThresholding` class, the following parameters are required:

- `omics_dataframe`: Omics data to be processed in a pandas dataframe format.
- `thresholding_strat`: String containing the thresholding strategy to be used. Must be one of: `global`, `local t1`, `local t2`.
- `global_threshold_lower`: Position of the Global Lower threshold value on the quantile list.
- `global_threshold_upper`: Position of the Global Upper threshold value on the quantile list.
- `local_threshold`: Position of the Local threshold value on the quantile list.

After creating the instance, the original dataset can be transformed with the `apply_thresholding_filter` method. This will return a new pandas dataframe with the TAS value for each gene for each sample.

```
threshold = GeneLevelThresholding(omics_dataframe=omics_data,
                                  thresholding_strat='local t2',
                                  global_threshold_lower=2,
                                  global_threshold_upper=3,
                                  local_threshold=2)

omics_data_after = threshold.apply_thresholding_filter()
omics_data_after

omics_data_after.T.describe()
```

2.3. Run FastCORE

A typical reconstruction workflow follows two main steps. The first is to attribute a score to each reaction of the model, in accordance with the omics data imputed. The second is to use the scores and apply an integration method to select a subset of reactions to build the final model.

The integration scoring methods implemented in *Troppo* are:

- `ContinuousScoreIntegrationStrategy`
- `ThresholdSelectionIntegrationStrategy`
- `DefaultCoreIntegrationStrategy`
- `AdjustedScoreIntegrationStrategy`
- `CustomSelectionIntegrationStrategy`

The omics integration methods implemented in *Troppo* are:

- `GIMME`
- `tINIT`
- `FASTCORE`
- `IMAT`
- `SWIFTCORE`
- `CORDA`

Note that the appropriate integration scoring method can differ between integration algorithms, but this pipeline can be easily adapted for other integration algorithms.

To simplify the process, and to allow for the integration of multiple samples using multiprocessing, it is possible to use the `run_from_omics` method from the `ReconstructionWrapper` class. This method first maps the gene IDs on the omics data to the gene IDs on the model and calculates scores for each reaction based on the Gene-Protein Rules. It then integrates those scores using custom or predefined functions, and finally runs the reconstruction algorithm selected.

To be able to run this function the following steps must be carried out:

Create a model wrapper

The `ModelBasedWrapper` class is used to wrap the model so that it can be used by *Troppo*.

Relevant arguments from this class include:

- `model`: The model to be wrapped.
- `ttg_ratio`: The ratio between the number of reactions to be selected and the total number of reactions in the model.
- `gpr_gene_parse_function`: A function that parses the GPRs of the model. This is used to map the identifiers in the omics data to the identifiers in the model.

Important attributes from this class include:

- `model_reader`: A `COBRAModelObjectReader` instance containing all the information of the model, such as reaction IDs, metabolite IDs, GPRs, bounds, etc.

- `S`: The stoichiometric matrix of the model.
- `lb`: The lower bounds of the reactions in the model.
- `ub`: The upper bounds of the reactions in the model.

In this specific example, we will use the `ReconstructionWrapper` class instead of the base `ModelBasedWrapper` class.

```
# Define the parsing rules for the GPRs that will be used on the wrapper.
patt = re.compile('__COBAMPGPRDOT__[0-9]{1}')
replace_alt_transcripts = lambda x: patt.sub('', x)

model_wrapper = ReconstructionWrapper(model=model, ttg_ratio=9999,
    gpr_gene_parse_function=replace_alt_transcripts)

model_wrapper
```

Create OmicsContainers Instances for Each Sample of the Omics Data

The `TabularReader` class is used to read and store the omics data in a container that can then be used by *Troppo*.

Relevant arguments from the `TabularReader` class include:

- `path_or_df`: The omics data can be either a pandas dataframe or a path to a dataset file. The file can be in any format supported by pandas.
- `index_col`: The name of the column that contains the identifiers of the genes.
- `sample_in_rows`: A boolean indicating whether the samples are in rows or columns.
- `header_offset`: The number of rows to skip before reading the header.
- `omics_type`: A string containing the type of omics data. This is used to select the appropriate integration method.
- `nomenclature`: A string containing the nomenclature of the identifiers in the omics data. This is used to map the identifiers to the identifiers in the model.

The `to_containers()` method returns a list of containers, one for each sample of the dataset.

```
omics_container = TabularReader(path_or_df=omics_data_after, nomenclature='entrez_id',
    omics_type='transcriptomics').to_containers()
omics_container
```

Parameters for the Reconstruction

In order to batch run the FastCORE algorithm, we need to define the parameters that will be used in the reconstruction function.

In this case, the parameters are:

- `Threshold`: The threshold to be used in the integration method.
- `Reconstruction_wrapper`: The model wrapper to be used.
- `Algorithm`: The algorithm to be used.

Since we previously performed gene-level thresholding on the omics data, the value of the threshold can be set to 0. This means that we will be selecting reactions whose genes have a positive TAS value.

```
parameters = {'threshold': 0, 'reconstruction_wrapper': model_wrapper, 'algorithm': 'fastcore'}
```

2.4. Reconstruction of Context-Specific Models Using `run_from_omics`

In order to better understand which parameters are required for the `run_from_omics` function, it is important to first consider the different steps it performs for each sample.

Create a Data Map

For this, the `get_integrated_data_map()` method from the `TabularReader` class is used. This maps the gene IDs in the omics dataset to reaction IDs in the model through their GPRs and attributes a score to each reaction in accordance with the expression values of the associated genes. This method returns a dictionary with the reaction IDs as keys and the scores as values.

Important arguments from this method include:

- `model_reader`: A `COBRAModelObjectReader` instance containing all the information of the model. It can be accessed through `model_wrapper.model_reader`.
- `and_func`: A function used to combine the scores of the genes associated with a reaction for AND rules in the GPR. In this example, we will use the minimum function, meaning that the score of a reaction with AND in its GPRs will be the minimum score of the genes associated with it.
- `or_func`: A function used to combine the scores of the genes associated with a reaction for OR rules in the GPR. In this example, we will use the sum function, meaning that the score of a reaction with OR in its GPRs will be the sum of the scores of the genes associated with it.

Integrate Scores

For FastCORE, a custom integration strategy will be used. The goal is to return a list of reactions whose calculated score is above the defined threshold.

While the `ThresholdSelectionIntegrationStrategy` class could achieve this, a custom strategy was selected to also include a set of reactions to be protected during the integration.

For this, the `CustomSelectionIntegrationStrategy` class will be utilized. This class allows the user to insert a custom function to integrate the scores. In this case, the custom function will be named `integration_fx`, and it must be passed to the `run_from_omics` method as the following tuple `('CustomSelectionIntegrationStrategy', [integration_fx])`.

Running the `FastCORE` Reconstruction Algorithm

The `run_from_omics` method will also require parameters for the reconstruction algorithm to be passed along. These will be divided into two sets of parameters: those for the properties class and those for the algorithm class. For the `FastCORE` algorithm, these are:

`FastcoreProperties`

- `core`: List of indexes of core reactions as determined by integrated scores.
- `flux_threshold`: Flux threshold for the algorithm.
- `solver`: Solver to be used.

`FASTcore`

- `S`: The stoichiometric matrix of the model, accessible via `model_wrapper.S`.
- `lb`: The lower bounds of the reactions, accessible via `model_wrapper.lb`.
- `ub`: The upper bounds of the reactions, accessible via `model_wrapper.ub`.
- `properties`: A `FastcoreProperties` instance containing the algorithm properties.

Note that these parameters will differ based on the selected algorithm.

Parameters Required for the `run_from_omics` Function

- `omics_data`: The omics data container for the sample.
- `algorithm`: A string specifying the algorithm for reconstruction.
- `and_or_funcs`: A tuple with functions for the AND and OR operations of the GPR.
- `integration_strategy`: A tuple with the integration strategy and the function to apply to the scores.
- `solver`: The solver for optimization.
- `**kwargs`: Additional parameters specific to the algorithm used.

Output

The `run_from_omics` method returns a dictionary containing all model reactions with their respective Boolean value, indicating whether the reaction is active in the model. Note that this function will run for each sample individually, hence the dictionary will only contain the results for one sample.

```
def reconstruction_function_fastcore(omics_container, parameters: dict):

    protected_reactions = ['biomass_human']

    def integration_fx(reaction_map_scores):
        return [[k for k, v in reaction_map_scores.get_scores().items() if (v is not None and
             v > threshold) or k in
                  protected_reactions]]


    threshold, rec_wrapper, method = [parameters[parameter] for parameter in
                                      ['threshold', 'reconstruction_wrapper', 'algorithm']]

    AND_OR_FUNCS = (min, sum)

    if method == 'fastcore':
            return rec_wrapper.run_from_omics(omics_data=omics_container, algorithm=method,
                 and_or_funcs=AND_OR_FUNCS ,
                                          integration_strategy=('custom',
                                               [integration_fx]) ,solver='CPLEX')
```

Note that in this case, we are defining a function that will use the `run_from_omics` method. This is because the goal is to apply a parallelization method to allow for multiple sample integration. If the goal were only to reconstruct a context-specific model for one sample, calling `rec_wrapper.run_from_omics()` would suffice.

2.5. Run in Batch

Using the `reconstruction_function_fastcore` defined above, we can run the FastCORE algorithm in batch for multiple samples. This can be achieved by using the `batch_run` function from *Cobamp*, which is compatible with *Troppo*.

For this, the required parameters are:

- `function`: The function to be parallelized.
- `sequence`: The sequence where the parallelized function is to be applied.
- `paramargs`: The specific parameters of the function.
- `threads`: The number of threads to use for parallelization.

```
batch_fastcore_res = batch_run(reconstruction_function_fastcore, omics_container, parameters,
    threads=2)
batch_fastcore_res

fastcore_res_dict = dict(zip([('fastcore', container.condition) for container in
    omics_container], batch_fastcore_res))
fastcore_df = pd.DataFrame.from_dict(fastcore_res_dict, orient='index')
fastcore_df
```

3. Conclusion

This study presents a robust pipeline for reconstructing context-specific metabolic models in healthy and COVID-19-infected tissues using bulk RNA-seq data and the FastCORE algorithm. By integrating transcriptomic data from the GTEx and GSE150316 datasets into the Human-GEM model, this framework systematically reconstructs context-specific models, enabling the exploration of metabolic alterations induced by SARS-CoV-2 infection. This approach facilitates targeted investigations into host metabolic reprogramming by the virus during infection. To ensure transparency and reproducibility, the datasets and implementation are publicly available, supporting further applications in metabolic modeling and precision medicine. This pipeline is part of a published work, for further details on the analyses performed, we recommend consulting the original publication [2].

References

[1] Niyati Desai, Azfar Neyaz, Annamaria Szabolcs, Angela R Shih, Jonathan H Chen, Vishal Thapar, Linda T Nieman, Alexander Solovyov, Arnav Mehta, David J Lieb, et al. Temporal and spatial heterogeneity of host response to sars-cov-2 pulmonary infection. *Nature communications*, 11(1):6319, 2020.

[2] Alexandre Oliveira, Miguel Rocha, and Oscar Dias. Metabolic modelling reveals key pathways in covid-19 in an effort to drive drug purposing. *IFAC-PapersOnLine*, 58(23):91–96, 2024.

[3] Anne Richelle, Chintan Joshi, and Nathan E Lewis. Assessing key decisions for transcriptomic data integration in biochemical networks. *PLoS computational biology*, 15(7):e1007185, 2019.

[4] Jonathan L Robinson, Pınar Kocabaş, Hao Wang, Pierre-Etienne Cholley, Daniel Cook, Avlant Nilsson, Mihail Anton, Raphael Ferreira, Iván Domenzain, Virinchi Billa, et al. An atlas of human metabolism. *Science signaling*, 13(624):eaaz1482, 2020.

[5] Leslie Sobin, Mary Barcus, Philip A Branton, Kelly B Engel, Judy Keen, David Tabor, Kristin G Ardlie, Sarah R Greytak, Nancy Roche, Brian Luke, et al. Histologic and quality assessment of genotype-tissue expression (gtex) research samples: A large postmortem tissue collection. *Archives of Pathology & Laboratory Medicine*, 2024.

[6] Nikos Vlassis, Maria Pires Pacheco, and Thomas Sauter. Fast reconstruction of compact context-specific metabolic network models. *PLoS computational biology*, 10(1):e1003424, 2014.
